# Engineering Oncogenic Hotspot Mutations on *SF3B1* via CRISPR-Directed PRECIS Mutagenesis

**DOI:** 10.1158/2767-9764.CRC-24-0145

**Published:** 2024-09-24

**Authors:** Mike M. Fernandez, Lei Yu, Qiong Jia, Xuesong Wang, Kevyn L. Hart, Zhenyu Jia, Ren-Jang Lin, Lili Wang

**Affiliations:** 1 Department of Systems Biology, Beckman Research Institute, City of Hope National Comprehensive Cancer Center, Monrovia, California.; 2 Irell & Manella Graduate School of Biological Sciences, Beckman Research Institute, City of Hope National Comprehensive Cancer Center, Monrovia, California.; 3 Department of Botany and Plant Sciences, University of California, Riverside, Riverside, California.; 4 Center for RNA Biology and Therapeutics, Beckman Research Institute, City of Hope, Duarte, California.; 5 Toni Stephenson Lymphoma Center, Beckman Research Institute, City of Hope Comprehensive Cancer Center, Duarte, California.

## Abstract

*SF3B1* is the most recurrently mutated RNA splicing gene in cancer. However, research of its pathogenic role has been hindered by a lack of disease-relevant cell line models. Here, our study compared four genome engineering platforms to establish *SF3B1* mutant cell lines: CRISPR-Cas9 editing, AAV homology-directed repair editing, base editing (ABEmax, ABE8e), and prime editing (PE2, PE3, PE5max). We showed that prime editing via PE5max achieved the most efficient *SF3B1* K700E editing across a wide range of cell lines. Our approach was further refined by coupling prime editing with a fluorescent reporter that leverages a *SF3B1* mutation-responsive synthetic intron to mark successfully edited cells. By applying this approach, called prime editing coupled intron-assisted selection (PRECIS), we introduced the K700E hotspot mutation into two chronic lymphocytic leukemia cell lines, HG-3 and MEC-1. We demonstrated that our PRECIS-engineered cells faithfully recapitulate known mutant *SF3B1* phenotypes, including altered splicing, copy number variations, and cell-growth defect. Moreover, we discovered that the *SF3B1* mutation can cause the loss of Y chromosome in chronic lymphocytic leukemia. Our results showcase that PRECIS is an efficient and generalizable method for engineering genetically faithful *SF3B1* mutant models. Our approach provides new insights on the role of *SF3B1* mutation in cancer and enables the generation of *SF3B1* mutant cell lines in relevant cellular context.

**Significance:**

This study developed an approach that can reliably and efficiently engineer *SF3B1* mutation into different cellular contexts, thereby revealing novel roles of *SF3B1* mutation in driving aberrant splicing, clonal evolution, and genome instability.

## Introduction

Advances in gene-editing technologies promise to revolutionize cancer research via precise and accurate modeling of specific point mutations (1). This is particularly relevant for the study of *SF3B1*, RNA splicing factors involved in the U2 snRNP recognition of the 3′ splice site (SS) during pre-mRNA splicing, that is recurrently mutated in multiple different leukemias and solid tumors (2). Hotspot mutations on *SF3B1* drive cryptic 3′ splice site selection, rewiring cellular splicing circuitries and promoting oncogenesis in a variety of cancers (3–7). Murine models with lineage-restricted expression of *Sf3b1* mutation have enabled mechanistic and functional dissection of the role of *SF3B1 in vivo* (8, 9); however, these models are restricted by high operating cost that limits the availability of different cancer models, human-to-mouse disease heterogeneity, and difficulty in further genetic manipulation. *In vitro* cell line systems are therefore an attractive orthogonal model for the functional and mechanistic dissection of *SF3B1* mutation owing to the ease of engineering and broad representation from across the cancer spectrum.

**Figure 1:**
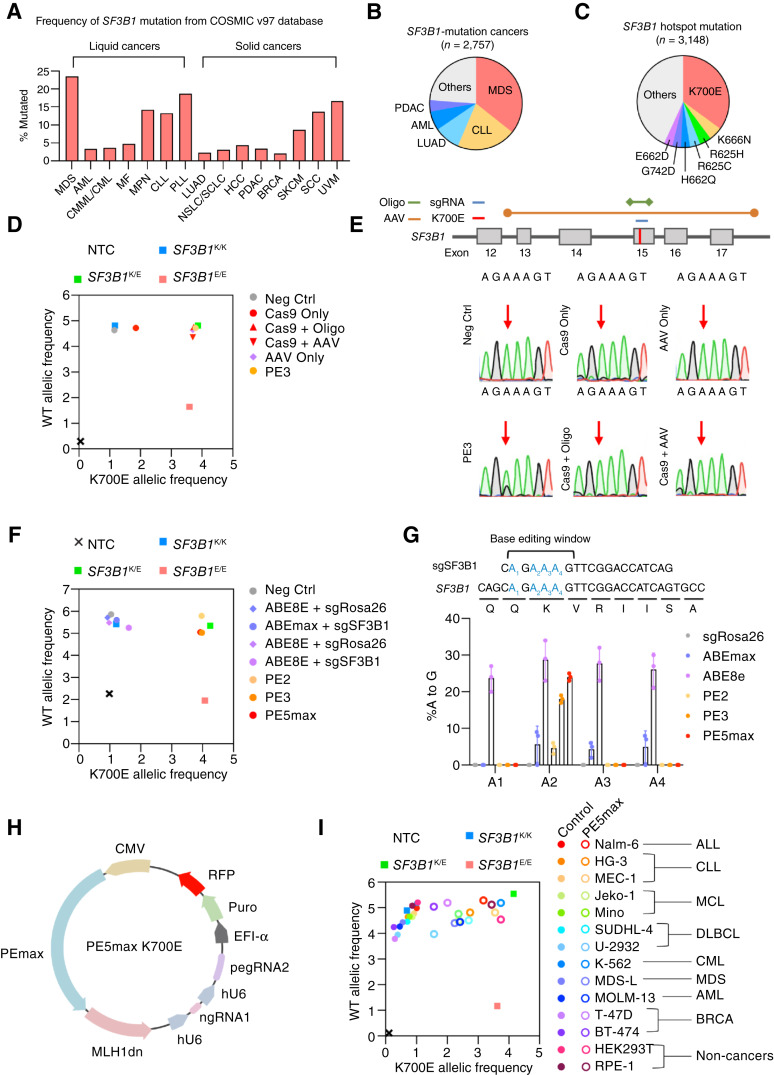
Prime editing can engineer the *SF3B1* mutation efficiently and across spectrum of cell lines and lineages. **A,** Frequency of *SF3B1* mutation across different cancers and malignancies based on COSMIC v97. All data were derived from the COSMIC database. **B,** All cancers and malignancies containing *SF3B1* mutation represented by proportion of total number of mutated cases from COSMIC v97. **C,** The most common *SF3B1* mutation with associated amino acid changes and frequency from COSMIC v97. **D,** Allelic discrimination plot comparing K700E editing for PE3 vs. Cas9 and AAV-homology-directed repair in HEK293T based on rhAMP SNP assay. **E,** Top, Scheme showing the editing strategies for inserting the K700E mutation using Cas9 and AAV HDR and (bottom) Sanger sequencing results from HEK293T transfected with indicated constructs. Red arrow indicates the nucleotide targeted for editing (AAA > GAA). **F,** Allelic discrimination plot comparing K700E editing for different prime editing systems vs. base editors in HEK293T based on rhAMP SNP assay. **G,** Top, Schematic for the K700 locus denoting the base editing window and the targetable adenosine positions colored in blue and denoted by subscript and (bottom) editing efficiency of experiment performed in **F** measured through Sanger sequencing in biological triplicates. **H,** The design of the PE5max K700E all-in-one construct. A cassette with pegRNA and ngRNA under hU6 promoters along with a downstream ORF containing puromycin selection marker, and TagRFP is cloned into a backbone containing PEmax and MLH1dn. **I,** Allelic discrimination plot showing PE5max editing of K700E in a variety of cell lines based on rhAMP SNP assay. All negative controls are parental, unedited cells. For all allelic discrimination plots, the square boxes indicate allelic reference controls: K/K (blue) is homozygous WT using K562 *SF3B1* WT gDNA; K/E (green) is heterozygous mutant using K562 *SF3B1* K700E gDNA; E/E (red) is homozygous mutant using pUC19-*SF3B1*-K700E plasmid. Some figures created with BioRender.com.

Based on the COSMIC v97 database, *SF3B1* is recurrently mutated in many different types of cancers with a particularly high frequency in hematological malignancies at the K700E position on exon 15 (Fig. 1A–C); refs. 10, 11) While several isogenic *SF3B1* mutant cell lines have been generated (Supplementary Table S1; refs. 12–15), the majority of these do not model the *SF3B1* mutation in the proper cancer contexts. For example, while *SF3B1* mutation occurs at high frequency in myelodysplastic syndrome (MDS) and acute myeloid leukemia (AML), the only isogenic *SF3B1* mutant myeloid cell line is in K-562, a chronic myelogenous leukemia (CML) cell line (4). Conversely, the pre-B ALL-derived Nalm-6 cell line (4) is the most commonly used B-cell line model for *SF3B1* mutation, despite *SF3B1* being recurrently mutated in chronic lymphocytic leukemia (CLL; ref. 16), a mature B malignancy.

The lack of proper isogenic models is attributable to several factors, with one key culprit being the limitations of current site-specific mutagenesis methods. Most *SF3B1* mutant cell lines are engineered via CRISPR-Cas9 or AAV technologies (3–5, 17, 18). Both approaches utilize homology-directed repair (HDR) to insert the K700E mutation into *SF3B1*, which relies on the cell’s intrinsic DNA repair capacity for efficient editing and underlies the limited availability of *SF3B1* mutant cell lines. Because *SF3B1* is essential to cell survival, engineering approaches that use double-stranded DNA breaks (DSB) as prerequisite to editing are severely penalized. This is further confounded by most cancer cell lines often exhibiting defective DNA repairs. Furthermore, efficient HDR is heavily dependent on *TP53* status that is frequently impacted across different tissues and cancers cell lines (19, 20). These factors all contribute to the limited availability of disease-relevant *in vitro* models.

Novel and emergent CRISPR-based technologies such as base editing and prime editing alter the genome through non-DSB approaches and, therefore, are less cytotoxic and more efficient than Cas9 HDR (21). Base editing pairs a nickase Cas9 (nCas9) with either a cytosine or adenosine deaminase to effectuate CRISPR-directed DNA editing in the genome (22). In this capacity, base editing has been proven effective in targeting splicing genes for mutagenesis (23). However, base editing suffers from poor mutagenic accuracy, rendering certain types of allelic edits impossible (24, 25). In contrast, prime editing effectuates genome-editing through CRISPR-directed reverse transcription (26, 27). A prime editing guide RNA (pegRNA) initiates editing by directing the nCas9 to nick single-stranded DNA that is then primed by the pegRNA for reverse transcription to incorporate edits into the genome. Consequently, prime editing is more accurate than base editing, while remaining less cytotoxic and more efficient than Cas9 and AAV-based approaches (26).

In this study, we show that prime editing can efficiently engineer the oncogenic *SF3B1* K700E hotspot mutation into cell lines, outperforming contemporary Cas9, AAV, and base editing approaches. We further show that prime editing can be coupled with a synthetic intron splicing reporter to facilitate cell line engineering that we used to create *SF3B1* mutant CLL cell lines. Finally, we demonstrate that these CLL *SF3B1* mutant cell lines accurately model for phenotypes of *SF3B1*-mutated CLL such as altered splicing and copy number variations, highlighting prime editing as a powerful tool for future pathogenic studies.

## Materials and Methods

### Molecular cloning

All primers used for cloning are listed in Supplementary Table S2. To generate a *SF3B1* K700E positive control for rhAMP SNP assays, a FragmentGene (GeneWiz) containing the K700E mutation (AAA > GAA) plus 350 bp of upstream and downstream sequences was amplified using Q5 polymerase (New England Biolabs) and cloned into pUC19 (Invitrogen) between HindIII and BamHI (New England Biolabs). The pUC19-*SF3B1*-K700E plasmid is also used as a double-stranded DNA (dsDNA) template for Cas9 HDR. All oligos for sgRNA cloning were synthesized by Integrated DNA Technology (IDT). All sgRNA and pegRNA sequences are listed in Supplementary Table S3. Cas9 HDR sgRNA sequence was designed using HDR Donor Designer (Horizon Discovery) and ligated into pSpCas9(BB)-2A-GFP (Addgene plasmid 48138) between BbsI (New England Biolabs) cut sites to produce the *SF3B1* sgRNA Cas9-GFP construct. Single-stranded DNA (ssDNA) oligo with *SF3B1* K700E mutation to serve as repair template for Cas9-HDR was generated through the Alt-R CRISPR service (IDTs). Base editing sgRNA directed against the *SF3B1* K700 locus was designed using BE-Designer (28) and cloned into pLKO.5 Puro-2A-GFP between BsmBI (New England Biolabs) cut sites. pegRNAs and ngRNAs were designed using PegIT (29). All pegRNAs were ligated into the pU6-pegRNA-gg-acceptor vector (Addgene plasmid 132777) between BsaI (New England Biolabs) cut sites as described (26). All ngRNAs were cloned into pLKO.5 Puro-2A-RFP between BsmBI (New England Biolabs) cut sites, resulting in the ngRNA1 or ngRNA2 pLKO.5 Puro-2A-RFP backbone. To construct the pCMV-PE5max-*SF3B1*-K700E vector, the hU6-pegRNA2-polyT cassette was amplified from the pU6-pegRNA-gg-acceptor backbone and ligated into ngRNA1 pLKO.5 Puro-2A-RFP between EcoRI and XhoI (New England Biolabs) to create pLKO.5-K700E-ng1+pg2-Puro-2A-RFP. The hU6-ngRNA1-hU6-pegRNA2-EF1-α-Puro-2A-RFP-WPRE cassette was amplified from this backbone and ligated into pCMV-PEmax-P2A-hMLH1dn (Addgene plasmid 174828) between SgrDI (Thermo Fisher Scientific) cut sites. To generate the pLenti-*SF3B1*-K700E-reporter, the *MTERF2* synthetic intron (synMTERFD3i1-150; ref. 30) was used to split EGFP between residues Q95 and E96 to create the EGFP synthetic intron reporter (EGFPi). An EF1-α promoter was used to drive the biscistronic expression of a blasticidin resistance marker (BSD) and EGFPi separated by a 2A autocleavable signal. This cassette was placed in an anti-sense orientation between two LoxP sequences and a WPRE signal and cloned into the pTwist+lenti+SFFV backbone (Twist Biosciences) between BamHI and XhoI (New England Biolabs) cut sites. The SFFV promoter is removed using Q5 site-directed mutagenesis (New England Biolabs). All plasmid constructs were verified using Primordium whole-plasmid sequencing (Primordium Labs).

### Human samples

Blood samples were drawn with written informed consent from patients enrolled in clinical protocol as approved by the Human Subjects Protection Committee of City of Hope in accordance with the Helsinki Declaration and other ethical guidelines. To isolate CLL B-cells, peripheral blood mononuclear cells were enriched through density gradient centrifugation followed by immuno-magnetic negative selection using the Pan B-cell isolation kit (MiltenyBiotec). Samples were then cryo-preserved until needed for study. The *SF3B1* mutation was verified in patient samples through a targeted sequencing panel.

### Cell lines and culture

HEK293T, RPE-1, and BT-474 cells were cultured in DMEM (Gibco) with 10% FBS (Omega Scientific) and 100 μg/mL penicillin-streptomycin (Gibco). T-47D and Nalm-6 were cultured in RPMI (Gibco) with 10% FBS (Omega Scientific) and 100 μg/mL penicillin-streptomycin (Gibco). HG-3, MEC-1, Jeko-1, Mino, SUDHL-4, U-2932, MDS-L, and MOLM-13 were cultured in RPMI (Gibco) with 20% FBS (Omega Scientific), 100 μg/mL penicillin-streptomycin (Gibco), and 2 mmol/L supplemented glutamine (Gibco). MDS-L was co-cultured with 30 ng/mL human IL-3 (Prepotech). K-562 was cultured in IMDM (Gibco) with 10% FBS (Omega Scientific) and 100 μg/mL penicillin-streptomycin (Gibco). BT-474 and T-47D were generously gifted by Dr. Chun-Wei Chen (City of Hope). RPE-1 was generously gifted by Dr. Jeremy Stark (City of Hope). MDS-L was generously gifted by Dr. Ling Li (City of Hope). MOLM-13 was generously gifted by Dr. Jianjun Chen (City of Hope). Nalm-6 and K-562 cell lines endogenously expressing *SF3B1* K700K or K700E were purchased from Horizon Discovery. HEK293T with endogenous expression of *SF3B1* K700E was generated through prime editing. HEK293T, HG-3, and MEC-1 WT isogenic controls are cells that do not have the K700E mutation but arose through single-cell cloning alongside the K700E clones. Adherent cells were maintained on six-well plates, while suspension cells were grown on 12-well plates. All cell lines were regularly tested and confirmed to be negative for mycoplasma.

### AAV and lentiviral packaging

AAV packaging and purification was done as described (31). The pAAV-SEPT-*SF3B1*-K700E donor plasmid and pRC1 and pHelper packaging plasmids were generously donated by Dr. Brian William Dalton (Johns Hopkins Medicine; ref. 3) and were transfected into HEK293T at 80% confluency in 10 cm plate in a 1:1:1 ratio of 10 μg each. Three days after transfection, the AAV packaging and purification were done as described (31). Briefly, AAV-*SF3B1*-K700E virions were released from HEK293T using the freeze-thaw method and precipitated overnight at 4°C using PEG 8000 (Fisher Scientific). Precipitates were pelleted via centrifugation at 2,800 *g* for 15 minutes at 4°C, resuspended in 2 mL PBS, and then incubated with 2 μL of DNase I (New England Biolabs), 2 μL RNase A (Invitrogen), and 3.5 μL of 1 mol/L MgCl_2_ (Invitrogen) for 20 minutes at 37°C. Viral supernatant was then mixed 1:1 with chloroform and incubated for 30 minutes at room temperature. After centrifuging at 3,000 *g* for 15 minutes at room temperature, the top aqueous layer was isolated and passed through an Amicron Ultra-0.5 filter (MilliporeSigma). PBS was passed through the column twice to wash the concentrated virus and to remove chloroform. The final concentrated viral was eluted from the column by inverting the column into a 1.5 mL tube and centrifuging at 1,000 *g* for 1 minute. For AAV HDR experiments, HEK293T were pre-seeded onto 12-well plates and grown to 80% confluency before transduction by addition of the concentrated virus to the media. For combination with Cas9 editing, HEK293T was transduced 24 hours before transfecting in *SF3B1* sgRNA Cas9-GFP. To engineer cell lines harboring the pLenti-*SF3B1*-K700E-reporter, cells were transduced via spin-infection. To package lentivirus, 3 μg of viral plasmid donor, 0.4 μg of VSV-G, and 1.5 μg psPAX2 were mixed with PEI MAX 40K (Polyscience) and transfected into seeded HEK293T in six-well format. Lentiviral supernatant was collected at 72 and 96 hours after transfection, pooled, and filtered through a 0.45 μm PES syringe filter (Bioland Scientific) before being concentrated with ultracentrifugation at 88,000 *g* for 2 hours at 4°C. To transduce suspension cells, 0.5 million cells were seeded into a 48-well format with 8 μg/mL polybrene and concentrated lentivirus and spin-infected at 1,000 *g* for 1.5 hours at 37°C. The media was changed 3 hours after the spin-transduction. Transduced cells were then selected with 10 μg/mL blasticidin (InvivoGen) for 5 days.

### Transfection and flow cytometry

For Cas9 and AAV HDR, base editing, and prime editing experiments in HEK293T cells, 50,000 cells were seeded onto 12-well plates, grown to 80% confluency, and transfected using PEI MAX 40K (Polysciences). For Cas9 and AAV HDR experiments, 1 μg of *SF3B1* sgRNA Cas9-GFP was transfected alone if cells were pre-transduced with AAV-*SF3B1*-K700E or with 0.1 nmole of the *SF3B1* Alt-R HDR template. For base editing experiments, 1 μg of either *SF3B1* sgRNA or *Rosa26* sgRNA in pLKO.5 Puro-2A-GFP backbone was co-transfected with either 1 μg of NG-ABE8e (Addgene plasmid 13849) or NG-ABEmax (Addgene plasmid 124163). For prime editing experiments, 0.5 μg of pegRNAs were co-transfected alone or with 0.5 μg ngRNA with 1 μg of either pCMV-PE2-P2A-GFP (Addgene plasmid 132776) or pCMV-PEmax-P2A-MLH1dn (Addgene plasmid 174828). HEK293T, RPE-1, T-47D, and BT-474 were transfected using PEI MAX 40K (Polysciences). For prime editing with the pCMV-PE5max-*SF3B1*-K700E all-in-one construct, 3 μg of the plasmid was transfected into HEK293T, RPE-1, T-47D, and BT-474 on six-well plates at 80% confluency. Nalm-6, HG-3, MEC-1, Jeko-1, Mino, SUDHL-4, U-2932, MDS-L, MOLM-13, and K-562 were electroporated using the Celetrix LE+ system (Celetrix). For electroporation, ∼30 to 40 million cells were resuspended in 200 μL volume of electroporation buffer and 30 μg of plasmids and electroporated at 1,100 V, 30 ms, 1× pulse. Cells were allowed to recover for 3 to 4 days before being taken for genomic DNA (gDNA) extraction. For prime editing experiments, gDNA from parental untransfected cells was used as negative control in line with previous works (26, 32). For testing Cas9 HDR in HG-3 cells, Alt-R Cas9v3 and *SF3B1* sgRNA RNP (IDTs) were assembled into RNPs following manufacturer’s instruction. Then, five million cells were electroporated with either 0.125 nmole of assembled RNP and 0.1 nmole of the *SF3B1* Alt-R HDR template or 10 μg of *SF3B1* sgRNA Cas9-GFP plasmid and 30 μg of the pUC19-*SF3B1*-K700E plasmid as a dsDNA repair template. HG-3 cells were electroporated using either Neon (Invitrogen) in the case of RNP or the Celetrix LE+ system (Celetrix) in the case of plasmid. For the former, the following settings were used to get >99% electroporation efficiency: 1,600 V, 10 ms, 3× pulses. For the latter, cells were electroporated using 1,100 V, 30 ms, 1× pulse followed by cell sorting using an Aria Fusion (BD Biosciences). To engineer *SF3B1* K700E cell lines via prime editing, ∼30 to 40 million cells harboring the pLenti-*SF3B1*-K700E-reporter were electroporated with 20 μg of the pCMV-PE5max-*SF3B1*-K700E and expanded more than 3 days before being sorted for RFP for which at least 200,000 events were collected. Sorted cells were re-seeded into pre-warmed media in a 96-well format and expanded more than 7 days before being sorted for GFP, collecting at least 200,000 events. Sorted cells were then re-seeded into pre-warmed media in a 96-well format and expanded for 4 days before being taken for single-cell cloning. Some cells were collected at day 7 after RFP sorting and day 4 after GFP sorting for gDNA isolation to check for presence of the K700E mutation. To remove pLenti-*SF3B1*-K700E-reporter, 20 μg of pCMV-mCherry-Cre (Addgene plasmid 27546) was electroporated into ∼30 to 40 million cells using the setting 1,100 V, 30 ms, and 1× pulse and sorted for RFP. Electroporated cells were recovered, expanded, and sorted again for GFP negative cells followed by single-cell cloning to isolate pure clones. All flow cytometry was performed on a Fortessa X20 (BD Biosciences), and flow data were analyzed using FlowJo version 10.8.2 (BD Biosciences).

### Cell cycle and cell proliferation assays

Cell cycle was performed with some modifications as previously described (33). In brief, 200,000 cells were seeded on 24-well plates with 1 mL of media and cultured overnight. The next day, cells were treated with a final concentration of 10 mmol/L EdU (Invitrogen) for 1 hour at 37°C. Cells were then resuspended in 100 μL fixation buffer (BioLegend) and incubated in dark for 15 minutes. Cells were then washed with 2 mL PBS buffer and incubated in 100 μL 1× saponin (Invitrogen) in dark for 15 minutes. A Click-IT reaction containing 2 mmol/L Cu_2_SO_4_, reducing reagent, and AF488 dye (Click Chemistry Tools) was added followed by an incubation period of 30 minutes in dark. Finally, DAPI was added, and cells were then taken for flow analysis on a Fortessa X20 (BD Biosciences). For cell proliferation assay, 20,000 cells were seeded onto 96-well plates in 200 μL final media volume and cultured overnight. The next day, 100 μL of suspended cells were incubated with 10 μL of CCK-8 reagent (Dojindo) for 3 hours at 37°C. Absorbance readings were taken using an Infinite M1000 Pro plate reader (Tecan) at 450 nm at 24, 48, and 72 hours post-seeding. Cell cycle and cell proliferation results were plotted using GraphPad Prism (Dotmatics).

### Genetic analysis

In experiments involving rhAMP SNP high-throughput screening of single-cell clones, gDNA was released from cells by thermolysis. In brief, cells were spun down in 96-well PCR plates (Bioland Scientific), resuspended in 20 μL water, and heated at 100°C for 10 minutes in a thermocycler (Applied Biosystems). After pelleting cellular debris, the supernatant was used for genotyping. For sequencing, gDNA was extracted from cells using the NucleoSpin Tissue kit (Macherey-Nagel). Parental, untransfected cells were used as negative controls in prime editing-related rhAMP SNP assays and sequencing. To check for the K700E mutation via Sanger sequencing or the Premium PCR NGS service (Primordium), the *SF3B1* K700 locus was amplified by PCR on gDNA using DreamTaq (Invitrogen) with the primer pairs: forward, 5′ CCTGTGTTTGGTTTTGTAGGTC 3′ and reverse, 5′ GGTGGATTTACCTTTCCTCT 3′. In the experiments in which presence of residual Alt-R HDR oligos or AAV-*SF3B1*-K700E DNA can result in false positives, PCR primers that only amplify gDNA were used: forward, 5′ GTGTAACTTAGGTAATGTTGGGGC 3′ and reverse, 5′ GAAGAGAAAAGTGACCAAACATCG 3′. PCR products were purified using the NucleoSpin Gel and PCR Clean-up kit (Macherey-Nagel) and sequenced using Sanger sequencing (Eton Biosciences) with the reverse primer for PCR done with the first primer pairs and the forward primer for PCR done with the second primer pairs. For Premium PCR sequencing (Primordium), samples were sequenced to a coverage depth of ∼4,400 reads per base pair. The Sanger sequencing trace files were aligned with the *SF3B1* gene using SnapGene (Dotmatics). Editing efficiency was calculated using ICE (Synthego) and EditR (http://baseeditr.com; ref. 34) on biological triplicates from Sanger sequencing. For NGS-based Premium PCR sequencing, allele frequencies were calculated from read counts. Deletion of the pLenti-*SF3B1*-K700E-reporter via Cre recombinase was confirmed with PCR using two pairs of primers: PCR1 - forward, 5′ CTTGCTCACCATCGGTCCAG 3′ and reverse, 5′ ATGGCCAAGCCTTTGTCTCAAG 3′; PCR2 - forward, 5′ ACTGCTGATCGAGTGTAGCC 3′ and reverse, 5′ TCATTGGTCTTAAAGGTACCGAGC 3′. PCR1 and PCR2 were performed at the annealing temperatures of 61°C and 58°C, respectively, with an extension time of 30 seconds at 72°C. Cre recombinase-mediated deletion of the K700E reporter was assessed on 2% agarose gel in 1xTAE buffer with a 1kB Plus Ladder (Invitrogen). RhAMP SNP primers (IDTs) were designed and used to genotype cells. For clean gDNA isolated using kit, ∼100 to 200 ng was used as inputs for the PCR reaction. For crude gDNA released through thermolysis, 4.2 μL of the supernatant was used. For experiments in which residual Alt-R HDR oligos or AAV-*SF3B1*-K700E DNA may result in false positives, the *SF3B1* K700 locus was first amplified with the *SF3B1* AAV primer set to generate purified PCR products for rhAMP SNP assay. For rhAMP SNP assay on PCR products, 10 pg of PCR products were used. For rhAMP SNP assay on gDNA, 100 ng of gDNA was used. 1 ng of pUC19-*SF3B1*-K700E and 100 ng of gDNA from K562 *SF3B1* K700K and K700E were used as controls for homozygous and heterozygous mutants and WT, respectively. RhAMP SNP primers were designed by IDT DNA (assay name CD.GT.WVSM3699.1). RhAMP SNP assay was carried out per manufacturer’s protocol on a QuantStudio 12K Flex (Applied Biosystems) in a 384-well format (USA Scientific). For PCR of Y chromosome genes, ∼100 ng of gDNA were used as inputs. GAPDH was used as an internal control. Allelic discrimination was plotted using GraphPad Prism (Dotmatics). All primers used in rhAMP SNP, sequencing, PCR, and genotyping are listed in Supplementary Tables S4 and S5.

### Splice variant qPCR

Total RNA was extracted using TRIzol (Ambion), while cDNA was generated using the High-Capacity cDNA Reverse Transcription Kit (Applied Biosystems) per manufacturer’s protocol. 5 ng of total cDNA was used as inputs for qPCR on the QuantStudio 12K Flex (Applied Biosystems). To calculate relative ratio of splice variants, The Ct values for the normal and alternatively spliced isoforms were inputted into the following formula: 2^ΔΔCt(Normal)−ΔΔCt(Aberrant)^. The results were graphed using GraphPad Prism (Dotmatics). For *MTERF2* intron 1 splicing analysis, 5 ng of total cDNA was used as inputs for PCR reactions using three primers: *MTERF1* Ex1, 5′ ACT​CCC​TGT​GCC​TTG​CTT​G 3′; *MTERF1* In1, 5′ CAT​TTT​GGG​CAT​GGA​ATC​TG 3′; *MTERF1* Ex2-3 R, 5′ ATG​GAC​TCA​TTC​TAT​CTT​ACA​GTC​TCT​CC 3′. All PCR products were resolved on 2% agarose gel in 1xTAE buffer with a 1kB Plus Ladder (Invitrogen). Primers to detect splice variants are listed in Supplementary Table S5.

### Splicing and differential gene expression analyses

PolyA mRNA was enriched from total RNA and constructed into a library for RNA-seq using the Stranded Total RNA Prep with Ribo-Zero Plus Kit (Illumina). cDNA libraries were sequenced using paired-end 150 bp on the Illumina NovaSeq 6000 platform at 50 million reads per sample. Reads were aligned to the GRCh38 reference genome using STAR. Splicing analysis was performed using rMATS (35). RNA-seq was carried on HG-3 and MEC-1 clones that have underwent Cre recombination to remove the K700E reporter and isolated through single-cell cloning. RNA-seq data for Nalm-6 and K562 *SF3B1* WT and mutant cell lines were downloaded from NCBI Gene Expression Omnibus under accession GSE72790. For primary CLL patient samples, 23 *SF3B1* WT and 13 *SF3B1* mutant samples were used for RNA-seq analysis and were previously deposited (33). For splice variant analysis in primary patient samples, FDR ≥0.05, abs(ΔPSI) ≥0.1, and read coverage for each junction ≥3 were used as statistical cutoffs. For each cell line, different reads cutoffs for each junction were used to normalize for differential total sample reads between cell lines: Nalm-6, ≥10; HG-3, ≥15; MEC-1, ≥18. rMATS output for CLL, Nalm-6, HG-3, and MEC-1 are shown in Supplementary Tables S6–S9. To obtain differential gene expressions (DEG), gene read counts were obtained from RNA-seq data using STAR –quantMode GeneCounts. R package DESeq2 (36) was used for DEG identification. *P* values were calculated by using the Wald test and adjusted by using the Benjamini–Hochberg algorithm. The significant expressed gene thresholds were (i) adjusted *P* value of <0.05 and (ii) Log_2_FC ≥1 and ≤−1. DEG results for HG-3 and MEC-1 are presented in Supplementary Tables S10 and S11.

### CNV analyses from RNA-seq and whole-genome sequencing

RNA-seq data were used to analyze for copy number variation (CNV) in HG-3, MEC-1, and Nalm-6 *SF3B1* WT and K700E cell lines using RNAseqCNV (37). For whole-genome sequencing (WGS), gDNA was isolated from cell lines using the NucleoSpin Tissue extraction kit (Macherey-Nagel). gDNA amount and quality were assessed using a NanoDrop (Thermo Fisher) and sequenced with Nebula Genomics at 3 to 18× coverage. The WGS fastq files were aligned to the GRCh38 reference genome using BWA-MEM to generate BAM files. Subsequently, each BAM file underwent sorting and indexing through SAMtools (38). Duplicate reads were identified and marked using the MarkDuplicates function in Picard (https://broadinstitute.github.io/picard/). SAMtools (38) were then employed to generate an index for each BAM file with marked duplicate reads. The entire genome was segmented into windows with a width of 10,000 bp. The read depth within each window was extracted using mosdepth (39), producing a BED file that captured window start, end, and depth information. CNV detection was performed using the R package cn.mops (40), comparing the mutant sample against the wild-type sample through referencecn.mops and calcIntegerCopyNumbers functions. The outcome is a CNV table detailing the start, end, and the observed CNVs. WGS data for patients with primary CLL were downloaded from CLLmap (41).

### Single nucleotide variants and INDEL analyses

The sorted WGS BAM files were converted into pileup format using SAMtools (38). For each cell line, the *SF3B1* WT and mutant pileup files were designated as normal and tumor samples, respectively. Single nucleotide variants and insertion and deletion (INDEL) events were called using the somatic mode by VarScan (42). Off-target sites for pegRNA2 and ngRNA1 were predicted using the IDT CRISPR-Cas9 guide RNA design checker tool (https://idtdna.com/site/order/designtool/index/CRISPR_SEQUENCE). The top 29 sites for pegRNA2 and ngRNA1 were selected for INDEL profiling. Each off-target site was expanded by 100 bp on both sides, generating the off-target region. Each INDEL start position was aligned to the off-target regions to identify INDELs occurring near the off-target sites. For WT samples, the off-target INDEL number was determined by the frequency of the germline INDELs. For the *SF3B1* mutant samples, the off-target INDEL number was determined by the combined frequency of germline and somatic INDELs.

### Microsatellite length calls

Genomic coordinates for 19 known microsatellite sites (43) were first converted from GRCh37 to GRCh38 via liftover (44). The cell line INDELs were aligned to each coordinate. Within each coordinate, the lengths of each 19 microsatellite sites of the WT samples were calculated by subtracting the number of deleted nucleotides in the germline INDELs from the reference nucleotide repeat number. Similarly, the microsatellite lengths of the *SF3B1* mutant samples were calculated by subtracting the number of deleted nucleotides in both germline and somatic INDELs from the reference nucleotide repeat number.

### Y chromosome gene expression and signature score

Calculation of the Y chromosome gene signature score was adapted from previous approaches (45, 46). In brief, gene expression raw count table, mutation summary table, and clinical outcome table for patients with CLL were downloaded from CLLmap (16). Male patients were extracted from the clinical outcome table. The *SF3B1* mutation status was annotated using the mutation summary table. A total of 311 WT and 84 male patients who were *SF3B1* mutated were identified. To define a Y chromosome gene signature, all genes located on the Y chromosome (*n* = 469) were extracted using the R package biomaRt (47). Candidate Y chromosome genes were selected based on a minimum expression (raw count >10) in more than 100 patient samples, resulting in a final list of Y chromosome genes (*n* = 43). The average TPM for the 43 Y chromosome genes was calculated for each patient with CLL, serving as the Y chromosome gene signature score. DEG analysis was then performed using DESeq2 (36), with *P* values calculated using the Wald test and adjusted via the Benjamini–Hochberg algorithm. The thresholds for significance cutoffs were (i) adjusted *P* value <0.05 and (ii) Log_2_FC ≥1 and ≤−1.

### Cancer distribution and frequency of *SF3B1* mutation

The cancer distribution and frequency of *SF3B1* mutation were downloaded from COSMIC v97 (cancer.sanger.ac.uk) on January 28, 2023. COSMIC data were filtered for point mutations on *SF3B1* (COSG68561) and for cancers with at least >2% of samples with *SF3B1* mutation. The percentage of samples with *SF3B1* mutation for each cancer was calculated and plotted using GraphPad Prism (Dotmatics). The total numbers of samples with *SF3B1* mutation for each cancer types and samples with a specific *SF3B1* mutation were plotted as pie charts using GraphPad Prism (Dotmatics). Venn diagrams were created using the Multiple List Comparator from MolBioTools (www.molbiotools.com/listcompare).

### Statistical methods

All statistical tests were calculated using either GraphPad Prism (Dotmatics) or R statistical packages. Error bars are shown to represent the mean of three independent replicates. The specific statistical tests done for each experiment are indicated in the associated figure legend. An unpaired *t*-test was performed to calculate *P* values when comparing only two groups. For multi-group comparisons, a two-way ANOVA was used instead. A one sample Wilcoxon signed rank test was performed for all RNAseqCNV results using log_2_FC = 0 as the hypothetical value. A Mann–Whitney test was performed when comparing multiple *SF3B1* WT and mutant primary CLL patient samples.

### Data availability

The pCMV-PE5max-SF3B1-K700E (Addgene plasmid 200317) and plenti-SF3B1-K700E reporter (Addgene plasmid 200318) are available on Addgene (www.addgene.org/Lili_Wang//). All RNA-seq results for the HG-3 and MEC-1 *SF3B1* WT and K700E cell lines are available at the Gene Expression Omnibus database (GSE231387).

## Results

### Prime editing can efficiently engineer the *SF3B1* K700E hotspot mutation

The initial prime-editing system, also known as PE2, pairs a prime-editing enzyme composed of a nCas9 and a reverse transcriptase with a pegRNA (26). PE3 improves upon PE2 by using an additional nicking guide RNA (ngRNA) to introduce a secondary nick following the initial edit to bias DNA repair toward incorporating the edit into the genome (26). We assessed the efficiency of PE2 and PE3 in engineering the K700E hotspot mutation by introducing A > G transition on the first letter in the AAA three-letter lysine codon (Supplementary Fig. S1A). We designed pegRNAs and ngRNAs directed against the K700 locus and measured the editing efficiency in HEK293T cells using rhAMP SNP assay and Sanger sequencing (Supplementary Fig. S1B–S1D and S2A). Using these methods, we observed the highest editing efficiency with the PE3 system via the pegRNA2 and ngRNA1 combination (Supplementary Fig. S2B and S2C). We further benchmarked PE3 against conventional Cas9 and AAV HDR approaches in which we observed PE3 introducing the K700E mutation at a higher frequency and lower INDELs (Fig. 1D and E). Encouraged by these results, next, we compared PE2 and PE3 to another iteration of the prime editing technology, PE5max. It is known that mismatch repair (MMR) is antagonistic to prime editing (32). Temporary inhibition of MMR can favor incorporation of edits without causing microsatellite instability (32). The PE5max improves prime editing by co-expressing an evolved prime editor (PEmax) with a dominant negative isoform of MLH1 (MLH1dn) to temporarily inhibit MMR (Supplementary Fig. S1C). In our tests, PE5max outperformed both PE2 and PE3 in installing K700E mutation on *SF3B1*, achieving ∼30% efficiency in HEK293T cells (Fig. 1F and G; Supplementary Fig. S2D and S2E). Given that *SF3B1* is tr-allelic in HEK293T and that only mono-allelic K700E mutation is possible, this result highlights the efficiency of prime editing.

Next, we compared prime editing to base editing, an orthogonal gene-editing technology. Unlike prime editor, base editors use adenosine deaminases to directly convert A > G on DNA in a sgRNA-dependent manner (48). We assessed two base editors, ABEmax and ABE8e, and compared their editing efficiency versus PE5max for *SF3B1* K700E editing in HEK293T cells. At the first adenosine position, PE5max attained A > G editing efficacy comparable to the two base editors, ABEmax and ABE8e (Fig. 1F and G; Supplementary Fig. S2D and S2E). However, the base editors also induced high levels of bystander edits to adjacent adenosines resulting in unintended missense mutations. Because the K700 and adjacent codons are adenosine-enriched, this locus is largely uneditable by base editors due the poor mutagenic accuracy, rendering prime editing as the only system that can engineer the K700E mutation. These results highlight the general specificity of prime editing over base editing and establish PE5max as the most optimal system for *SF3B1* engineering.

While prime editing works remarkably well in HEK293T in which MMR is deficient, its effectiveness in other cell lines in which MMR is intact can be lacking (27, 32). Considering that inclusion of the MLH1dn MMR inhibitor may allow us to efficiently engineer the K700E mutation in a wider variety of cellular contexts, we first reconstituted the components of PE5max—pegRNA2, ngRNA1, PEmax, and MLH1dn—into an all-in-one construct with puromycin and RFP selection markers (pCMV-PE5max-*SF3B1*-K700E, henceforth referred to as PE5max K700E; Fig. 1H). As a functional test of the PE5max K700E system, we used it to derive a pure isogenic model of *SF3B1* mutation in HEK293T (Supplementary Fig. S2F). Through electroporation or transfection of the PE5max K700E construct, we next assayed for K700E prime editing in a panel of cell lines from diverse cancers such as acute lymphoblastic leukemia (ALL), chronic lymphocytic leukemia (CLL), mantle cell lymphoma (MCL), diffused large B-cell lymphoma (DLBCL), chronic myelogenous/myelomonocytic leukemia (CMML), MDS, AML, breast cancers (BRCA), as well as non-cancer cell lines HEK293T and RPE-1 (Fig. 1I). While the untransfected parental cells closely clustered with the WT control, all PE5max K700E-treated cells shifted with varying degrees, likely reflecting differences in *SF3B1* zygosity and transfection/electroporation efficiency (Fig. 1I). This result overall indicates that *SF3B1* K700E engineering is possible across diverse cellular context via PE5max prime editing.

### Coupling prime editing with a *SF3B1* mutation-responsive reporter enables facile engineering

As a proof-of-concept, we next sought to use this system to address the dearth of *SF3B1* mutant B-cell lines. While *SF3B1* mutations occur in both lymphoid and myeloid malignancies, most available *SF3B1* mutant cell lines are in the myeloid lineage with few existing lymphoid models (Supplementary Table S1). *SF3B1* is recurrently mutated in CLL, a mature lymphoid B-cell cancer (11). In our initial effort to engineer the CLL cell line HG-3 with the *SF3B1* mutation using CRISPR Cas9 technology, we observed no successful cases of editing either using Cas9 ribonucleoprotein (RNP) and single-stranded DNA oligo (ssDNA; Supplementary Fig. S3A and S3B) or with Cas9 plasmid and a double-stranded DNA (dsDNA) repair template (Supplementary Fig. S3C and S3D). In the latter case, we also observed several instances of false positives resulting from random integration of the repair template (Supplementary Fig. S3C and S3D), thereby demonstrating the pitfalls of Cas9 HDR in engineering the *SF3B1* K700E mutation. We thus turned to prime editing to generate CLL *SF3B1* mutant cell line models using two CLL cell lines with pre-existing CLL-associated cytogenetic lesions: HG-3 (*del(13q)*, 50% of CLL cases; ref. 16) and MEC-1 (*del(17p)*, 20% of CLL cases; ref. 16). Following PE5max K700E electroporation, we could detect the K700E mutation using rhAMP SNP assay but not Sanger sequencing (Supplementary Fig. S3E and S3F). This result aligns well with our previous observation that prime editing could be less efficient in certain cellular contexts, such as mature B-cells that are known for being difficult to engineer (Fig. 1I; ref. 49). The low efficiency of editing increases the difficulty and lowers the chances of isolating pure K700E clones (Supplementary Fig. S3G). We next sought a solution to increase the abundance of edited cells.

**Figure 2:**
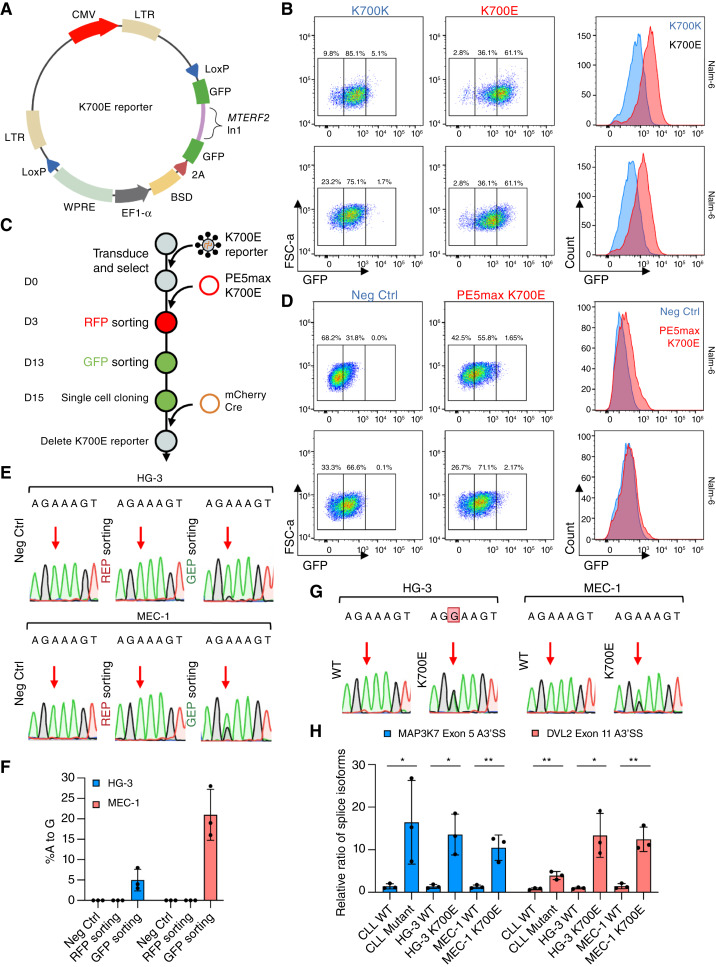
*SF3B1* mutation-responsive reporter allows for facile isolation of *SF3B1* mutant clones. **A,** The design of the K700E reporter. GFP is bisected by synMTERFD3i1-150 (30) and co-expressed with a blasticidin selection marker. The entire cassette is flanked by loxP signals to enable Cre recombinase-mediated deletion of reporter post-editing. **B,** Flow cytometry plots (left) and histograms (right) of Nalm-6 and K562 *SF3B1* K700K and K700E cell lines stably transduced with the K700E reporter. **C,** Workflow for engineering cell lines with the K700E mutation. **D,** Flow cytometry plots (left) and histograms (right) of HG-3 and MEC-1 control cells or cells electroporated and sorted for PE5max K700E. **E,** Sanger sequencing results for HG-3 and MEC-1 control cells, cells sorted for RFP (PE5max K700E), and cells sorted for GFP (K700E reporter). **F,** K700E frequency measured through Sanger sequencing for each sample in biological triplicates. **G,** Sanger sequencing results for isogenic HG-3 and MEC-1 *SF3B1* WT and K700E clones. **H,** Splice variant qPCR analysis of *MAP3K7* and *DVL2* alternative splicing in CLL, HG-3, and MEC-1 with and without *SF3B1* mutation in biological triplicates. Student unpaired *t*-test was used (*, *P* ≤ 0.05; **, *P* ≤ 0.01). All negative controls are parental, unedited cells. Some figures created with BioRender.com.

A recent report has shown that the intron 1 of the *MTERF2* gene splices out in a *SF3B1* K700E-dependent manner (Supplementary Fig. S3H; ref. 30). We verified this phenomenon in a panel of *SF3B1* K700E cell lines (Nalm-6, K-562, and HEK293T) in which we observed full *MTERF2* intron 1 removal only in mutant cells, while WT cells showed either full intron retention or partial intron splicing at one of the cryptic 3′SS (Supplementary Fig. S3I). This observation led us to hypothesize that this intron could be leveraged as a reporter for the K700E mutation. To test this, we inserted a previously validated, minimal *MTERF2* intron 1 (synMTERD3i1-150; ref. 30) between EGFP exons to create a fluorescent minigene reporter (EGFPi; Fig. 2A). The EGFPi cassette was placed into a lentiviral construct next to a blasticidin resistance gene (BSD) under the control of an EF1-α promoter. LoxP sites were included to allow for Cre recombinase-mediated deletion of this cassette. We tested this pLenti-*SF3B1*-K700E-reporter (henceforth referred to simply as the K700E reporter) in Nalm-6 and K-562 cells harboring either WT or *SF3B1* K700E mutation (Fig. 2B). The presence of the K700E mutation led to an increase in both GFP expression and the proportion of GFP bright cells in both cell lines. Given that this reporter can fluorescently label K700E prime edited cells, we devised an innovative approach termed PRECIS that pairs fluorescent sorting with prime editing (Fig. 2C; Supplementary Fig. S3J).

Using the reporter, we observed a noticeable increase in the GFP dim and bright populations in PE5max K700E-electroporated HG-3 and MEC-1 cells when compared with negative control cells (Fig. 2D; Supplementary Fig. S4A and S4B). Following FACS-enrichment of the GFP bright population, we observed detectable levels of A > G edits in HG-3 (∼5%) and MEC-1 (∼22%) through Sanger sequencing (Fig. 2E and F; Supplementary Fig. S4C and S4D). Clonal selection via single-cell cloning paired with high-throughput rhAMP SNP screening yielded HG-3 and MEC-1 clones harboring heterozygous K700E alleles (Fig. 2G; Supplementary Fig. S4E and S4F). For HG-3, we were able to derive pure heterozygous clones, while with MEC-1 only aneuploid (tri-allelic) clones were possible. We then removed the K700E reporter using Cre-loxP system through overexpression of Cre recombinase followed by another round of single-cell cloning to derive pure clones. (Supplementary Fig. S5A–S5D). We further confirmed the phenotypic impact of *SF3B1* mutation by checking for known alternative splicing in these cell lines including full *MTERF2* intron 1 splicing and upregulation of *MAP3K7* and *DVL2* splice variants (Fig. 2H; Supplementary Fig. S6A and S6B; ref. 7). Both mutant cell lines also showed defective G_1_-S phase transition and slower growth, consistent with previous findings of increased mitotic stress in *SF3B1* mutant cells (Supplementary Fig. S6C–S6E; refs. 7, 8, 50). These results confirmed that we have generated *bona fide* CLL *SF3B1* K700E cell lines.

### CLL *SF3B1* K700E cell lines share some common splice variants with *SF3B1*-mutated primary CLL

**Figure 3:**
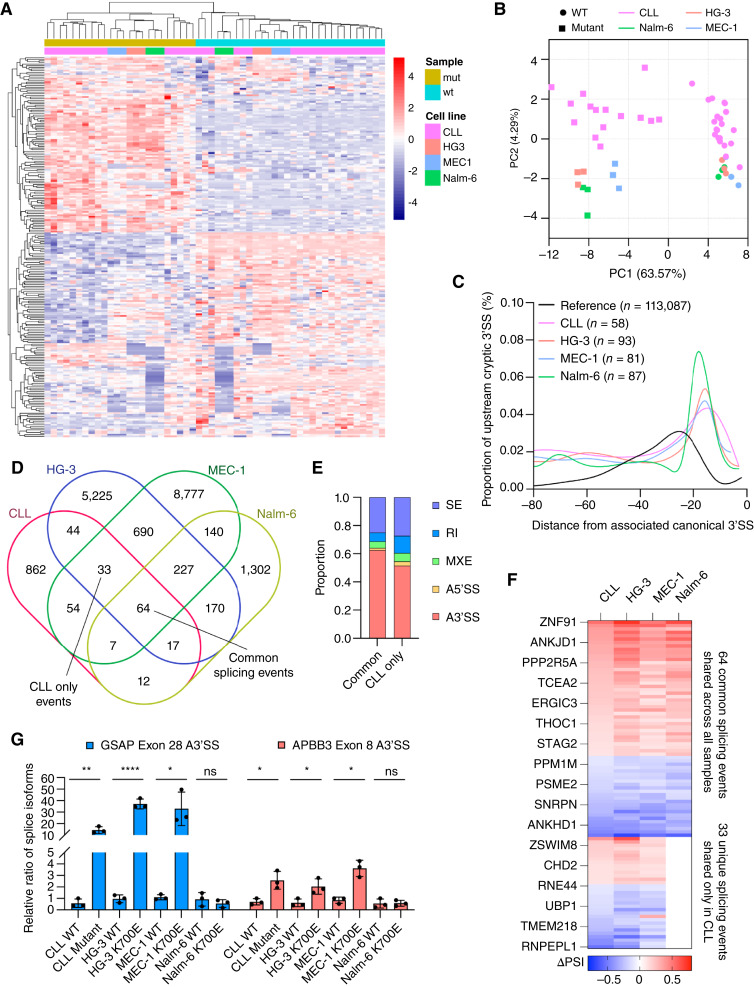
CLL *SF3B1* mutant cell lines recapitulates the altered splicing profile of primary CLL. **A,** Unsupervised clustering analysis and (**B**) PCA plot based on alternative splicing events of RNA sequencing data derived from primary CLL patient samples (*n* = 36) and cell lines with and without *SF3B1* mutation. **C,** Density plot showing the position and frequency of cryptic 3′SS for *SF3B1*-mutated primary CLL samples and cell lines vs. average distance to the first AG from RefSeq reference of all canonical 3′SS. The number of cryptic 3′SS events used to calculate each density plot is indicated. **D,** Overlap analysis of significant splicing events associated with *SF3B1* mutation (PSI > 0.1 or < −0.1 with *P* value < 0.05) among CLL, HG-3, MEC-1, and Nalm-6 cells. Events common to all samples or specific only to CLL and CLL cell lines are indicated. **E,** Stacked bar plot representation of the type of alternative splicing events found in common and CLL only splice variants. **F,** Heatmap showing the splicing profiles of 64 common and 33 CLL only splice variants. **G,** Splice variant qPCR analysis of *GSAP* and *APBB3* alternative splicing in CLL, HG-3, MEC-1, and Nalm-6 with and without *SF3B1* mutation in biological triplicates. Student unpaired *t*-test was used (*, *P* ≤ 0.05; **, *P* ≤ 0.01). Some figures created with BioRender.com.

We next sought to compare the splicing pattern of these novel CLL *SF3B1* mutant cell lines to Nalm-6 and primary *SF3B1*-mutated CLL cells. Across primary CLL samples, CLL cell lines, and Nalm-6, presence of *SF3B1* mutation drove a uniform splicing paradigm with all *SF3B1*-mutated samples clustering together (Fig. 3A and B). The presence of *SF3B1* mutation promoted selection of cryptic 3′SS proximal to within 15 nts of the canonical 3′SS across all samples in agreement with previous observations (Fig. 3C; Supplementary Fig. S7A; refs. 7, 51). Mutant *SF3B1* also preferentially affected alternative 3′SS splice site selections, as well as intron retention compared with other splicing events (Supplementary Fig. S7B). These results confirmed that the HG-3 and MEC-1 *SF3B1* K700E cell lines shared many similarities with *SF3B1*-mutated primary CLL and Nalm-6 cells. When we compared splice variants associated with the mutation, however, we found both common splice variants shared with both Nalm-6 and primary CLL cells, as well as novel splice variants shared only with primary CLL cells (Fig. 3D). Splice variants belonging to both common and CLL only groups were predominantly alternative 3′SS splicing events (Fig. 3E). Common splice variants showed common splicing patterns across all *SF3B1*-mutated samples, confirming a uniformity in *SF3B1* mutation-mediated splicing program, while CLL only variants are present in primary CLL and CLL cell lines (Fig. 3F). We confirmed the aberrant splicing of two CLL only targets that undergo alternative 3′SS splicing, *GSAP* and *APBB3*, in CLL and CLL cell lines (Fig. 3G). Altogether, these results show that HG-3 and MEC-1 *SF3B1* K700E cell lines can recapitulate some of the altered splicing of *SF3B1*-mutated CLL.

### *SF3B1* mutation drives general and CLL-specific CNV events

In CLL, *SF3B1* mutation frequently co-occurs with two common CLL genetic lesions, *del(13q)* and *del(17p)*, that result in ablation of the *BCL2*-targeting *MIR15*/16 locus and *TP53*, respectively (16, 41, 52). From the study of the clonal evolutionary history of CLL, these two genetic lesions are known to be founding clonal events, while *SF3B1* mutations are often subclonal events (16, 53). Because of this, *SF3B1* mutation is often suggested to promote CLL progression rather than initiation (11). In rare cases, CLL may progress to a type of B-cell lymphoma called Richter Syndrome that is characterized by notably higher levels of CNVs than compared with the antecedent CLL (41, 54, 55). Interestingly, these CNV events often exist downstream of Richter-transformed *SF3B1* mutant CLL, suggesting that *SF3B1* mutation drives clonal evolution via CNV changes (41, 54, 55). Large-scale clonal hematopoiesis studies have also provided supporting evidence for *SF3B1* mutation and CNV changes in potentiating oncogenesis (56, 57). Finally, in previous works from our group, we also found evidence for *SF3B1* mutation-driven genomic instability in both murine models and cell lines (9, 50). Therefore, we hypothesize that our cell lines can be used to model, to a certain degree, the CNV clonal evolution of *SF3B1*-mutated CLL.

**Figure 4:**
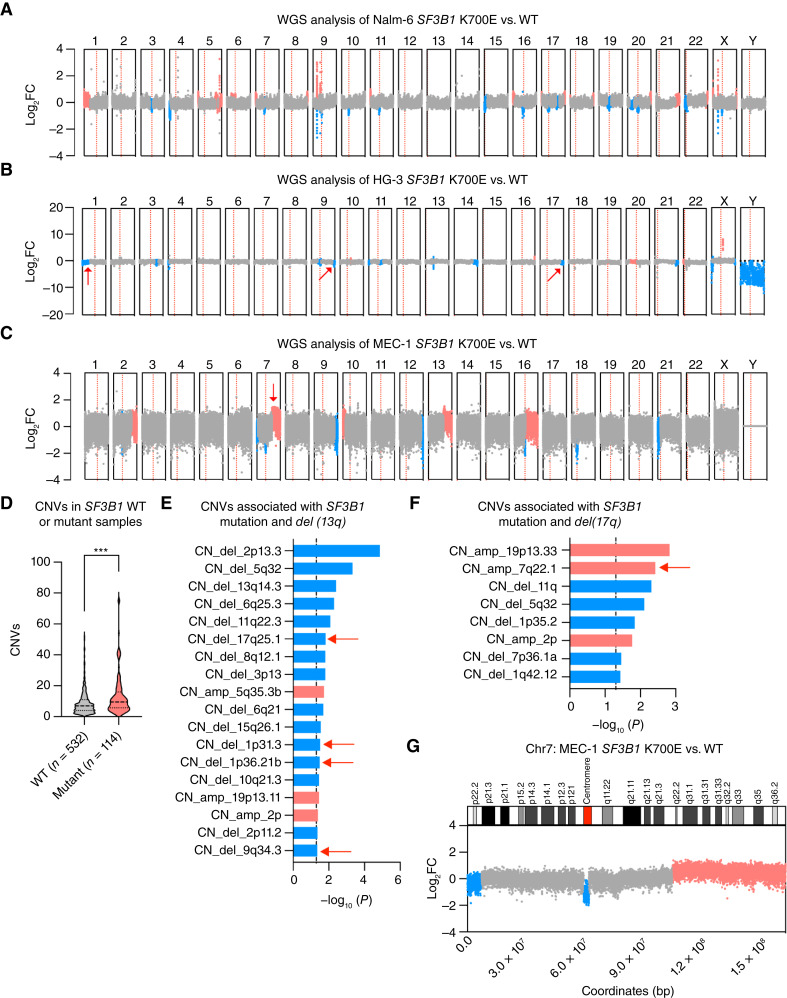
*SF3B1* mutant cells exhibit significant CNV changes that are consistent with primary CLL. CNVs analysis comparing *SF3B1* mutant samples vs. isogenic WT based on whole-genome sequencing data for (**A**) Nalm-6, (**B**) HG-3, and (**C**) MEC-1. Significant CNV events are denoted by blue for deletions and red for amplifications. Centromeric region demarcating the p and q chromosomal arms is indicated by a red dotted line. Red arrows indicate CNV events shared with primary patient CLL. **D,** Violin plot of the number of CNV events found in patients with *del*(17p) and *del*(13q) CLL that are either *SF3B1* WT or mutant based on CLLmap data (https://cllmap.org/). A Mann–Whitney test was used (***, *P* ≤ 0.001). CNVs associated with either (**E**) *SF3B1* mutation and *del*(13p) and (**F**) *SF3B1* mutation and *del(17p)* co-occurrences. The red arrow indicates chromosomal amplification shared between primary CLL samples and cell lines. A χ^2^ test was performed with −log_10_(0.05) as the statistical cutoff. **G,** Top, Cytogenetic loci on the p and q arm and (bottom) WGS analysis for chromosome 7. Some figures created with BioRender.com.

We first used a tool called RNAseqCNV (37) to infer the CNV status in our *SF3B1* WT and mutant cell lines. Across all cell lines, we were able to detect a few significant CNV events associated with *SF3B1* mutation, hinting that *SF3B1* mutation may play a conserved role in driving the acquisition of these events (Supplementary Fig. S8A–S8C). Supported by these initial observations, we next carried out whole-genome sequencing (WGS) of all *SF3B1* WT and mutant cell lines. Similar with our RNAseqCNV results, WGS revealed widespread genetic deletions and amplifications that tend to localize near telomeric and centromeric regions (Fig. 4A–C). Through WGS, we confirmed our initial RNAseqCNV results for significantly occurring CNV events such as a deletion of chromosome 4p in Nalm-6 (Fig. 4A; Supplementary Fig. S8A). CLL cell line HG-3 contained high prevalence of focal, rather than arm level, genetic deletions and amplifications that were invisible to RNAseqCNV but detectable through high resolution WGS (Fig. 4B; Supplementary Fig. S8B). In contrast, MEC-1 was characterized more by large-scale genetic changes at the chromosomal arm level (Fig. 4C; Supplementary Fig. S8C). Higher level of CNV events also appear to be associated with *SF3B1* mutation in primary CLL patient samples with either *del(13q)* or *del(17p)*, the two most common genetic lesions in CLL (Fig. 4D; refs. 16, 41, 52), suggesting that CNV changes are a general consequence of *SF3B1* mutation.

Next, we sought to determine what clinically relevant CNVs, if any, are shared between our cell lines and primary CLL patient samples. In the HG-3 cell line, which contains the pre-existing *del(13q)* genetic lesion, we identified genetic deletions at chromosomal locations 17q25.1, 1p31.3, 1p36.21b, and 9q34.3 shared with CLL patient samples containing both *SF3B1* mutation and *del(13q)* (Fig. 4E). In the MEC-1 cell line, which contains the pre-existing *del(17p)* genetic lesion, amplification at chromosome 7q22.1 stood as the most significant CNV event shared with patients with primary CLL harboring both *SF3B1* mutation and *del(17p)* (Fig. 4F). Nalm-6, in contrast, shared almost no common CNV events with CLL. These results demonstrate that the CLL *SF3B1* mutant cell lines recapitulate some CLL-specific CNVs. To confirm this further, we focused on amplification of chromosome 7q [*amp(7q)*] found in our MEC-1 cell line. Amplifications on chromosome 7 are frequent CNV events in CLL and a common downstream CNV event of *SF3B1* mutation following transformation of CLL to Richter Syndrome (16, 55). Breaking down WGS and RNAseqCNV calls for chromosome 7 in MEC-1, we confirmed arm level amplification of chromosome 7q starting at q22.1 (Fig. 4G; Supplementary Fig. S8D). These results collectively show that general and lineage-specific CNV events are hallmarks of *SF3B1* mutation-driven clonal evolution.

### *SF3B1* mutation drives loss of Y chromosome

**Figure 5:**
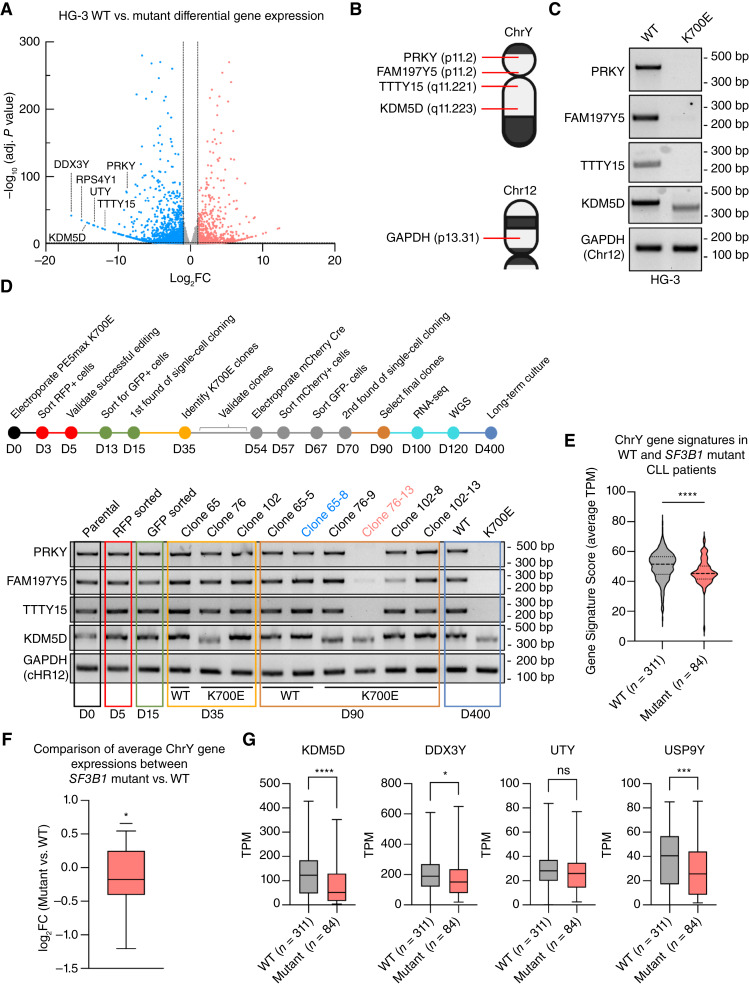
*SF3B1* mutation induces LOY in both cell lines and CLL patient samples. **A,** DEG analysis of HG-3 *SF3B1* mutant vs. WT cell lines. Log_2_FC ≥1 and ≤−1 and adjusted *P* value <0.05 chosen as statistical cutoffs. **B,** Schematic depiction of chromosome (top) Y and (bottom) 12 with PCR targets and loci indicated. **C,** PCR of Y chromosome genes in genomic DNA of HG-3 *SF3B1* WT and mutant cell lines. **D,** Top, Timeline for the PRECIS engineering of *SF3B1* K700E and (bottom) PCR of Y chromosome genes in HG-3 genomic DNA at different time points in the PRECIS engineering process. Clones 65-8 (blue) and 76-13 (red) became the final isogenic HG-3 *SF3B1* WT and mutant cell line, respectively. **E,** Y chromosome gene signature score for 43 Y chromosome genes in *SF3B1* WT and mutant male CLL patient samples. A Mann–Whitney test was used (***, *P* ≤ 0.001). **F,** DEG analysis of 43 Y chromosome genes comparing *SF3B1* mutant vs. WT male CLL patient samples. One-tailed *t*-test was performed with log_2_FC ≥0 as the null hypothesis and log_2_FC < 0 as the alternative hypothesis. **G,** TPM expression for different Y chromosome genes in *SF3B1* WT and mutant male CLL patient samples. Mann–Whitney test was used (****, *P* ≤ 0.0001; ***, *P* ≤ 0.001; *, *P* ≤ 0.05). Some figures created with BioRender.com.

Through RNAseqCNV and WGS analyses, we also discovered another phenotype in our CLL cell lines: The loss of Y chromosome (LOY). LOY, defined as the partial or full deletion of the Y chromosome, is previously thought to be a benign age-related event but is now known to be widespread in multiple cancers including leukemias (45, 58). In CLL, LOY is a major risk factor (59). However, its linkage with known genetic drivers is not well-characterized. Previous studies of risk factors in clonal hematopoiesis of indeterminate potential (CHIP) demonstrated a strong co-occurrence of *SF3B1* mutation and LOY (60, 61). We therefore focused on detecting LOY in our *SF3B1* mutant CLL cell lines. Combining both RNAseqCNV and WGS, we observed LOY in HG-3 *SF3B1* mutant but not WT cells (Fig. 4B; Supplementary Fig. S8B). Both *SF3B1* WT and mutant MEC-1 cell lines had LOY, suggesting that LOY was a pre-existing CNV in MEC-1 (Fig. 4C; Supplementary Fig. S8C). We thus focused our studies in HG-3 cells. In line with LOY detected by RNAseqCNV and WGS, differential gene expression (DEG) analysis showed a significant global downregulation of Y chromosome genes in HG-3 *SF3B1* mutant cells (Fig. 5A), highlighting a critical role of *SF3B1* mutation in driving LOY in CLL.

To verify this finding, we selected four genes that are distributed across the Y chromosome on both the P and Q arms to benchmark the degree of LOY in our cell lines (Fig. 5B). Using PCR-based approaches, we detected widespread LOY including deletion of the known tumor suppressor, *KDM5D*, in which loss resulted in aberrant PCR amplification of *KDM5C*, a *KDM5D* paralog on the X chromosome (ref. 62; Fig. 5C). Using this approach, we also verified LOY was a pre-existing CNV in the parental MEC-1 cell line (Supplementary Fig. S8E). Next, we sought to determine when and to what extent LOY occurred in our HG-3 cell lines. Through profiling gDNA saved from every step of the cell line engineering, we were able to observe the first LOY event, a *KDM5D* deletion, occurring at day 35 in the K700E clone 76 after the first round of single-cell cloning (Fig. 5D). Selected WT (clone 65) and mutant (clones 76 and 102) clones would undergo Cre recombinase treatment to remove the K700E reporter, which is then followed by a second round of single-cell cloning. The *KDM5D* deletion was carried into the second round of single-cell cloning, appearing in the clone 76 subclones (Fig. 5D). Clone 76-13, which was selected to be the final HG-3 *SF3B1* mutant cell line, acquired additional deletions at three other Y chromosomal loci (Fig. 5D). We also detected a partial *FAM197Y5* deletion in K700E clone 102-8, which suggested that while the degree of deletions can vary, LOY is a general *SF3B1* mutation phenotype (Fig. 5D; Supplementary Fig. S8F).

Given these findings, we examined the co-occurrences of LOY and *SF3B1* mutation in primary patient CLL. Using CLLmap data (16), we adapted previously established methods (45, 46) to calculate a Y chromosome gene signature score for each male CLL patient samples with and without *SF3B1* mutation (Fig. 5E). Compared with WT samples, presence of *SF3B1* mutation showed a significantly lower Y chromosome score (*P* ≤ 0.001, Mann–Whitney test), indicating a higher probability of LOY (Fig. 5E). DEG for Y chromosome genes further validated an overall decrease of Y chromosome gene expression in *SF3B1* mutant CLL samples (Fig. 5F). Individually, some of the most downregulated Y chromosome genes included known tumor suppressors such as *KDM5D* and *DDX3Y* (Fig. 5G; refs. 46, 63). These results are highly suggestive of a causal relationship between *SF3B1* mutation and LOY in CLL. Finally, to rule out the possibility that these CNV events occurred because of the use of a MMR inhibitor to increase prime editing efficiency, we first queried 17 microsatellite sites known to be truncated in cells with MMR defects for any signs of genetic instability (43). Consistent with previously published works (32), we observed no significant changes at these 17 sites based on WGS result of our CLL cell lines (Supplementary Fig. S9A). Then, we examined off-targeting of Cas9 by screening for INDELs at 29 predicted pegRNA and ngRNA off-target sites (Supplementary Fig. S9B and S9C). There were no significant levels of INDELs detected across all sites between WT and mutant CLL cell lines, reflecting previous observations of lower off-targeting for prime editing versus Cas9 (Supplementary Fig. S9B and S9C; refs. 21, 64). Finally, we also found that the majority of single nucleotide variants and INDELs were associated with *SF3B1* mutation, confirming that genetic perturbations were likely because of the K700E mutation and not gene editing (Supplementary Fig. S9D and S9E). These results collectively highlight the role of *SF3B1* mutation in driving downstream clonal changes, including novel CNV events such as LOY, and suggesting that LOY is a conserved mutant *SF3B1* phenotype in CLL.

## Discussion

Despite *SF3B1* being the most recurrently mutated splicing gene in cancers, there exist remarkably few isogenic cell lines modeling for *SF3B1* mutation in the appropriate disease contexts. Difficulties in engineering *SF3B1* have impeded further research about the roles of oncogenic mutations on this critical splicing factor. This study established a novel, precise, and highly efficient method for introducing *SF3B1* mutation at the endogenous gene locus across multiple cellular contexts based on prime editing technology. This proof-of-concept approach not only provides a generalizable approach for isogenic cell line engineering but also reveals novel aspects of cancer biology.

First, our all-in-one prime editing approach outperforms conventional Cas9 and AAV template-mediated editing, as well as base editing technology. We demonstrated that this system can introduce K700E mutation into cell lines that model MDS (MDS-L), AML (MOLM-13), and CLL (HG-3 and MEC-1). While our approach uses plasmids for cell line engineering, delivery of the prime-editing system using mRNAs and synthetic sgRNAs may potentially simplify and improve editing especially in hard-to-edit cell types in which electroporation of large cargos can be tricky. The use of ribonucleoproteins (RNP) and even viral platforms as delivery methods may even allow for highly efficient *SF3B1* engineering in primary cells such as CD34+ HSPCs, expanding the study of *SF3B1* mutation into primary human contexts. With the recent establishment of a prime-editing PE2 murine model, future *in vivo* prime edited models of mutant *SF3B1* and even other splicing factors can also be rapidly established (65).

Second, the use of synthetic intron reporter systems marking *SF3B1* mutant versus WT cells offers opportunities for further genetic engineering and screening. This manuscript showed that selection of edited cells with the K700E reporter significantly improved the efficiency of establishing *SF3B1* mutant cell lines. As the mutation responsiveness of the *MTERF2* synthetic intron is not limited to the K700E mutation, engineering possibilities may also be expanded to include other hotspot mutations such as the H662Q mutation (Supplementary Fig. S3H; ref. 30). With future discovery of additional mutation-responsive introns, prime editing may also be used to engineer hotspot mutations on other splicing factors such *SRSF2*, *U2AF1*, *ZRSR2*, genes encoding the U1 snRNA, and *HNRNPH1* (13).

Finally, we also showed that *SF3B1* mutation-associated CNV changes are a general *SF3B1* mutation signature in CLL. Building on previous works from our group (9, 50), through *in vitro* models, we demonstrated that *SF3B1* mutation gives rise to potentially clinically relevant CNVs such as *amp(7q)* and LOY. LOY is particularly interesting as it has been previously shown to be a risk factor in CLL (59). Yet, how LOY can contribute to CLL pathogenesis is unclear. In bladder cancer, LOY is shown to result in inactivation of tumor suppressors such as *KDM5D*, leading to cancer immune evasion and T-cell exhaustion (46). T-cell exhaustion is a known hallmark of CLL (66, 67), while *SF3B1* is among the most frequently mutated genes in CLL (>20%, depending on cohorts; Fig. 1A). These results support a model in which *SF3B1* mutation drives LOY as part of the clonal evolution of CLL, affecting CLL pathogenesis by interfering with both CLL intrinsic processes and inducing T-cell dysregulation. Additionally, while the overall genetic determinants of LOY remain under investigation, our work provides evidence that *SF3B1* mutation is a potential driver of LOY. Indeed, other works on CHIP have also linked occurrences of *SF3B1* mutation with increased LOY frequencies (60, 61). However, these findings still leave open the question of the mechanism underpinning *SF3B1* mutation-driven LOY development. As LOY can happen independently of *TP53* mutations (45), general genome instability alone cannot explain LOY development. GWAS studies have indicated an association between cell cycle defects and increased LOY frequency (68). Consistent with this, ours and other studies have shown cycle defects as being a *SF3B1* mutation phenotype, suggesting one possible mechanism for *SF3B1* mutation-induced LOY (Supplementary Fig. S6C and S6D; refs. 7, 8). Defects at the centromere can also result in chromosome missegregation and Y chromosome loss (69); we previously showed that *SF3B1* mutation-associated R-loop formations on centromeres drive mitotic defects and aberrant chromosomal segregation (50). These results point toward *SF3B1* mutation-driven aberrant mitosis and chromosomal instability as putative drivers of LOY.

Our results also leave additional unanswered questions that can be addressed in future studies. MEC-1 is previously known to contain an intact Y chromosome that is now lost in our culture (Supplementary Fig. S8E; ref. 70), highlighting a *SF3B1* mutation-independent LOY. The impact of LOY alone and LOY-*SF3B1* mutation co-occurrence would need to be investigated in large patient cohort studies to discern their respective contributions and synergism, if any. Additionally, Nalm-6 is also a male-derived cell line,yet its Y chromosome is intact even after long-term culture (Fig. 4A; Supplementary Fig. S8A). Our results indicate that LOY appears at an increasing frequency with single-cell cloning steps (Fig. 5D). As single-cell cloning is inherently stressful, cell stress could be one driver of LOY although the degree of LOY is inconsistent across our clones (Fig. 5D; Supplementary Fig. S8F), hinting toward additional confounding factors such as the broader tumor microenvironment that cannot be captured by our limited cell line model alone. In future studies, the PRECIS method could be paired with single-cell RNA and DNA sequencing to map out the *in vivo* clonal trajectory of *SF3B1* mutant cells, identify the development of CNVs such as LOY, and establish casual relationships that could be used to inform therapeutic responses to pharmacological inhibitors.

Taken as a whole, this study demonstrated a proof-of-concept approach that is a reliable, efficient, and cost-effective way to engineer *SF3B1* K700E mutation into diverse cell lines with broad implications for cancer research and drug development.

## Supplementary Material

Supplementary Figure 1 Overview of the K700 locus and mutation validation strategies

Supplementary Figure 2 PE2 and PE3 outperform base editing in engineering the K700E mutation

Supplementary Figure 3 Engineering SF3B1 mutation into CLL cell lines necessitates an additional enrichment marker

Supplementary Figure 4 FACS sorting and RhAMP SNP screening yield isogenic SF3B1 mutant clones

Supplementary Figure 5 The K700E reporter is removed via Cre-mediated recombinase treatment

Supplementary Figure 6 Splicing and growth phenotypes of HG-3 and MEC-1 SF3B1 mutant cell lines

Supplementary Figure 7 Aberrant 3’SS splicing profiles of SF3B1-mutated cell lines and primary CLL

Supplementary Figure 8 RNAseqCNV analysis uncovers widespread CNV events in SF3B1 mutant cell lines

Supplementary Figure 9 Off-targeting analyses for PRECIS engineering of SF3B1 K700E mutation

Supplementary Table 1 List of current SF3B1 mutant cell lines

Supplementary Table 2 Cloning and mutagenesis primers

Supplementary Table 3 PegRNA and sgRNA spacers

Supplementary Table 4 RhAMP SNP and sequencing primers

Supplementary Table 5 PCR and genotyping primers

Supplementary Table 6 CLL rMATS splicing output

Supplementary Table 7 Nalm-6 rMATS splicing output

Supplementary Table 8 HG-3 rMATS splicing output

Supplementary Table 9 MEC-1 rMATS splicing output

Supplementary Table 10 HG-3 DEG results

Supplementary Table 11 MEC-1 DEG results
