# Supplementary material for: Engineering Oncogenic Hotspot Mutations on SF3B1 via CRISPR-Directed PRECIS Mutagenesis
Source: Cancer Res Commun. 2024 Sep 24;4(9):2498–513. doi: 10.1158/2767-9764.CRC-24-0145 (PMC11421219; doi:10.1158/2767-9764.CRC-24-0145)
Supplement: Supplementary Figure 1 — Overview of the K700 locus and mutation validation strategies [file crc-24-0145_supplementary_figure_1_suppsf1.pdf]

Supplementary Figure 1

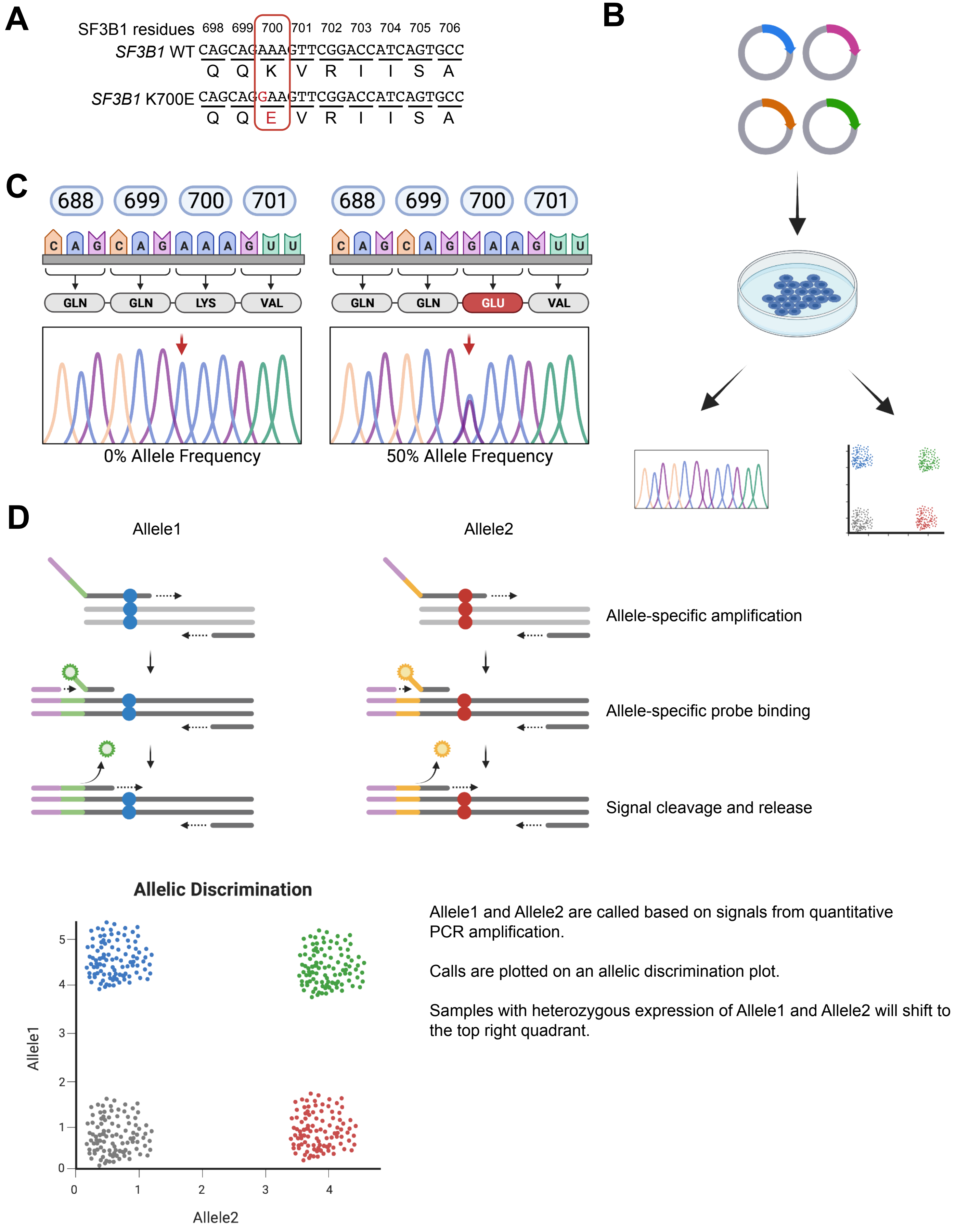

Supplementary Figure 1: Overview of the K700 locus and mutation validation strategies

A) The *SF3B1* K700 amino acid residue is encoded by a tri-nucleotide AAA sequence. Conversion of the first A>G will result in the hotspot K700E mutation. B) Workflow for testing prime editing of the K700E mutation. After transfecting, the prime editing components into HEK293T, the K700E editing efficiency is assessed via Sanger sequencing and rhAMP SNP assay. C) Cartoon representation of the expected Sanger sequencing trace for the K700 locus. The *SF3B1* K700E mutation is always heterozygous, reaching at most 50% allele frequency in diploid cells. D) Overview of the rhAMP SNP assay; Amplification by allele specific primers results in release of fluorescent signals that can be mapped on an allelic discrimination plot to give a readout on the presence of a SNP in each sample.
