# Supplementary material for: Engineering Oncogenic Hotspot Mutations on SF3B1 via CRISPR-Directed PRECIS Mutagenesis
Source: Cancer Res Commun. 2024 Sep 24;4(9):2498–513. doi: 10.1158/2767-9764.CRC-24-0145 (PMC11421219; doi:10.1158/2767-9764.CRC-24-0145)
Supplement: Supplementary Figure 2 — PE2 and PE3 outperform base editing in engineering the K700E mutation [file crc-24-0145_supplementary_figure_2_suppsf2.pdf]

Supplementary Figure 2

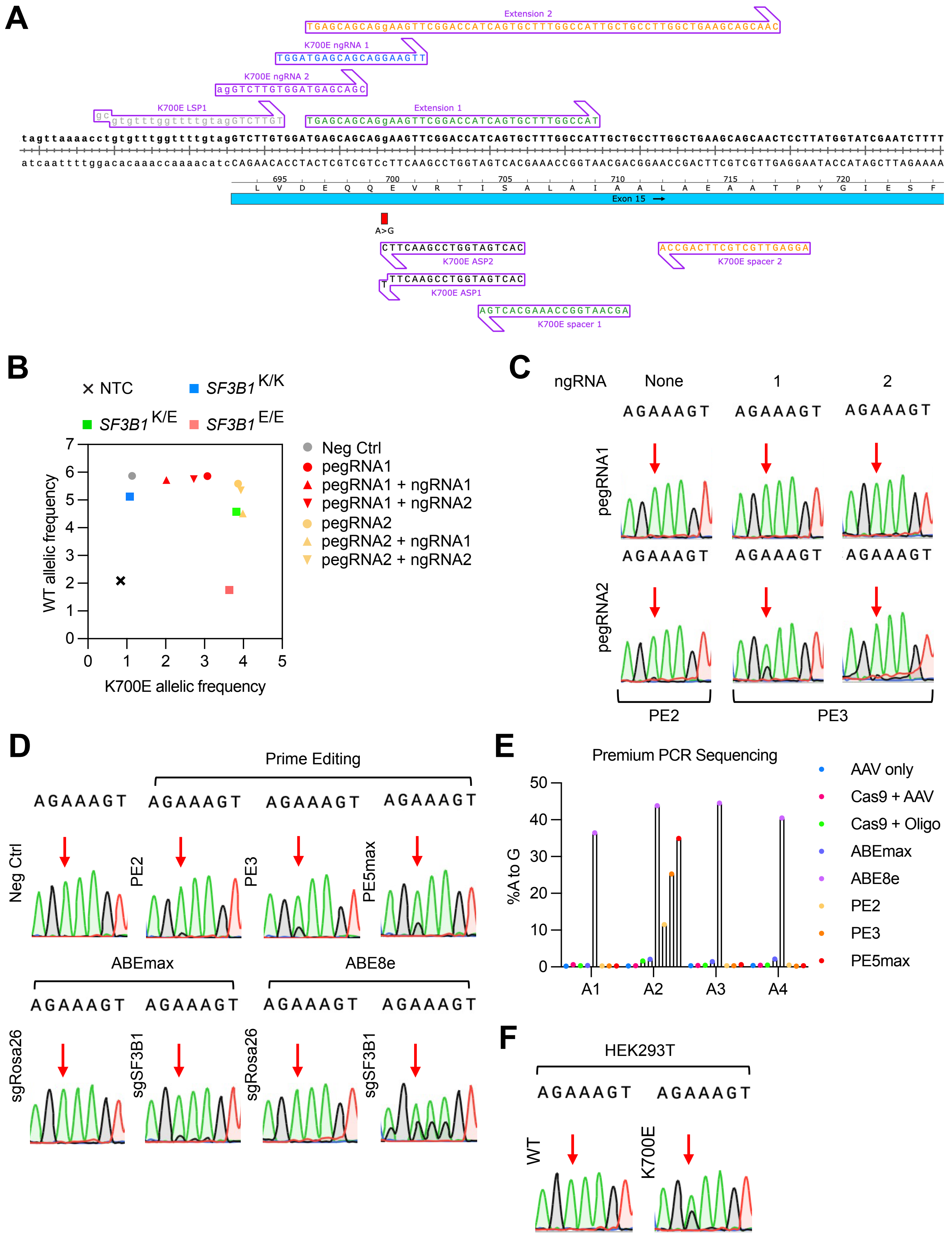

Supplementary Figure 2: PE2 and PE3 outperform base editing in engineering the K700E mutation

A) SnapGene view of *SF3B1* exon 15 with the locations and sequences of all pegRNAs and ngRNAs. RhAMP SNP primers are also indicated. B) Allelic discrimination plot showing prime editing of the K700E mutation using different pegRNAs alone or in combination with ngRNAs based on rhAMP SNP assay. C) Sanger sequencing results for HEK293T transfected with pegRNAs (PE2) alone or in combination with ngRNAs (PE3). D) Sanger sequencing results for prime editing versus base editing in HEK293T at the K700 locus. E) NGS-based Premium PCR sequencing results for editing at the K700 allele. F) Sanger sequencing results for isogenic HEK293T *SF3B1* WT and K700E clones. All negative controls are parental, unedited cells. For all allelic discrimination plots, the square boxes indicate allelic reference controls: K/K (blue) is homozygous WT using K562 *SF3B1* WT gDNA; K/E (green) is heterozygous mutant using K562 *SF3B1* K700E gDNA; E/E (red) is homozygous mutant using pUC19-*SF3B1*-K700E plasmid.
