# Supplementary material for: Engineering Oncogenic Hotspot Mutations on SF3B1 via CRISPR-Directed PRECIS Mutagenesis
Source: Cancer Res Commun. 2024 Sep 24;4(9):2498–513. doi: 10.1158/2767-9764.CRC-24-0145 (PMC11421219; doi:10.1158/2767-9764.CRC-24-0145)
Supplement: Supplementary Figure 3 — Engineering SF3B1 mutation into CLL cell lines necessitates an additional enrichment marker [file crc-24-0145_supplementary_figure_3_suppsf3.pdf]

Supplementary Figure 3

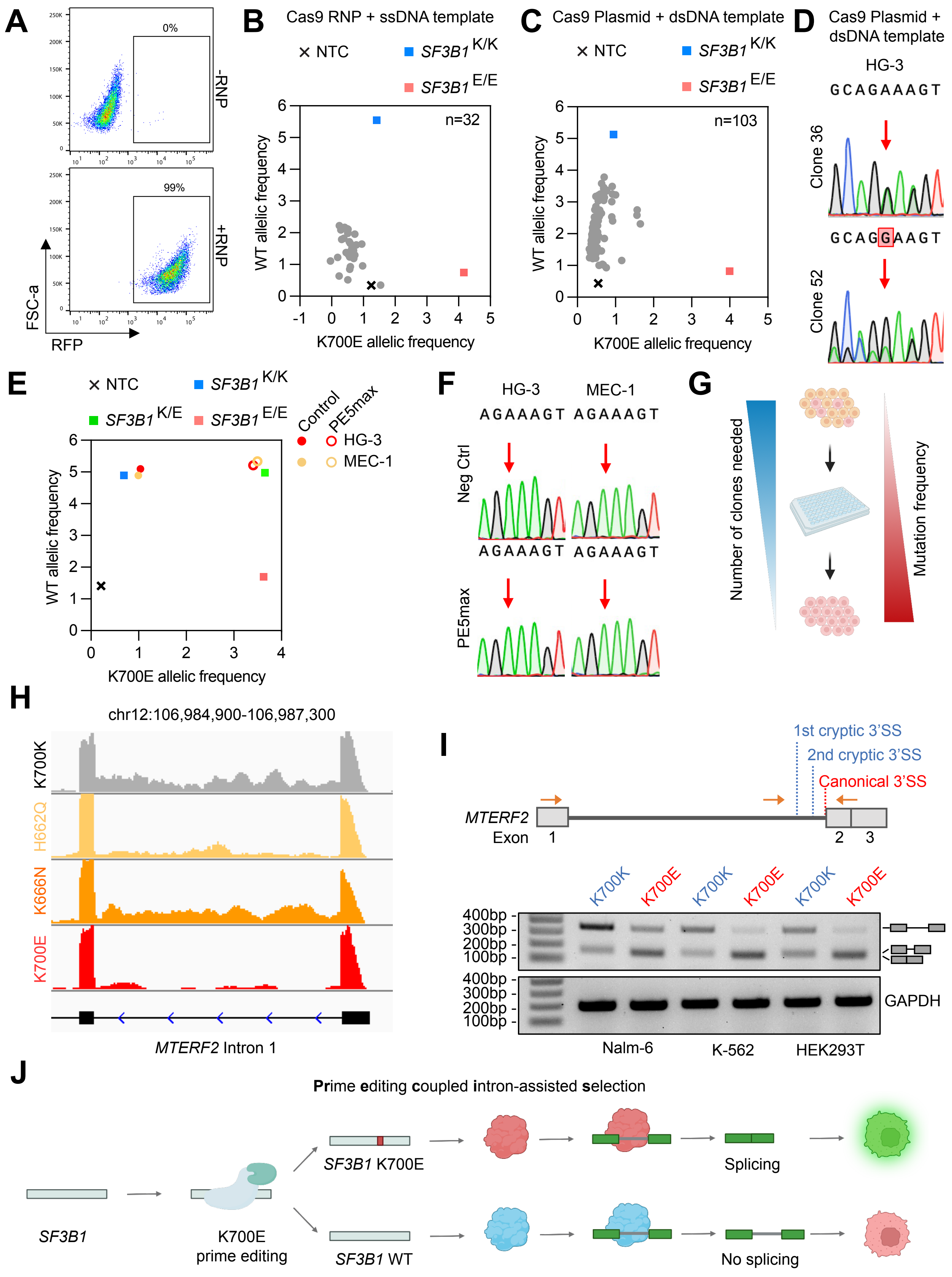

Supplementary Figure 3: Engineering *SF3B1* mutation into CLL cell lines necessitates an additional enrichment marker

A) Flow cytometry plots showing the electroporation of Cas9 RNP and ssDNA into HG-3. Color indicator is provided by ATTO-550 conjugated to the tracRNA. Allelic discrimination plot for single cell clones of HG-3 cells electroporated with B) Cas9 RNP and ssDNA or C) Cas9-GFP plasmid and dsDNA repair template. GFP is used as an indicator to sort for cells electroporated with plasmids before single cell cloning. D) Sanger sequencing results for two single cell clones isolated from HG-3 cells electroporated with Cas9-GFP plasmid and dsDNA repair template. E) Allelic discrimination plot and F) Sanger sequencing for prime editing by PE5max K700E in HG-3 and MEC-1 cells. G) For single cell cloning, higher efficiency editing in bulk cell populations will result in less clones needing to be screened to isolate pure *SF3B1* K700E clones. H) Nalm-6 RNA-seq reads for the *MTERF2* intron 1 splicing between isogenic WT and different *SF3B1* mutant clones. I) (top) Primer designs and (bottom) PCR for checking the splicing status on *MTERF2* intron 1 in different *SF3B1* WT and K700E cell lines. J) Overview of the PRECIS workflow: After the PE5max K700E is used to introduce the K700E mutation into cells, mutant *SF3B1* will splice the K700E reporter to give GFP expression as a marker for prime edited cells. All negative controls are parental, unedited cells. For all allelic discrimination plots, the square boxes indicate allelic reference controls: K/K (blue) is homozygous WT using K562 *SF3B1* WT gDNA; E/E (red) is homozygous mutant using pUC19-*SF3B1*-K700E plasmid.
