# Supplementary material for: Engineering Oncogenic Hotspot Mutations on SF3B1 via CRISPR-Directed PRECIS Mutagenesis
Source: Cancer Res Commun. 2024 Sep 24;4(9):2498–513. doi: 10.1158/2767-9764.CRC-24-0145 (PMC11421219; doi:10.1158/2767-9764.CRC-24-0145)
Supplement: Supplementary Figure 4 — FACS sorting and RhAMP SNP screening yield isogenic SF3B1 mutant clones [file crc-24-0145_supplementary_figure_4_suppsf4.pdf]

Supplementary Figure 4

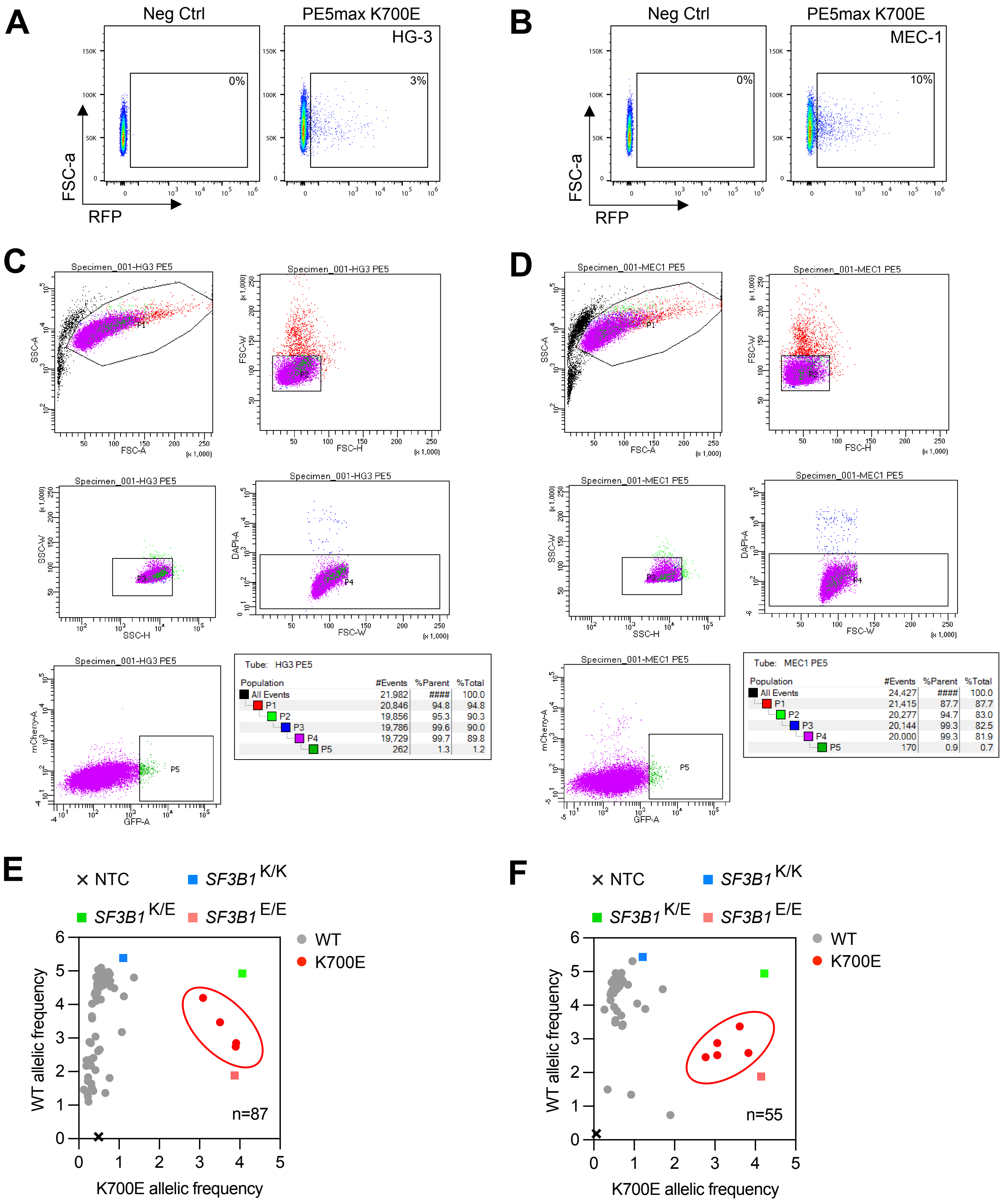

Tube: HG3 PE5

Population	#Events	%Parent	%Total
All Events	21,982	####	100.0
P1	20,846	94.8	94.8
P2	19,856	95.3	90.3
P3	19,786	99.6	90.0
P4	19,729	99.7	89.8
P5	262	1.3	1.2

Tube: MEC1 PE5

Population	#Events	%Parent	%Total
All Events	24,427	####	100.0
P1	21,415	87.7	87.7
P2	20,277	94.7	83.0
P3	20,144	99.3	82.5
P4	20,000	99.3	81.9
P5	170	0.9	0.7

Supplementary Figure 4: FACS sorting and RhAMP SNP screening yield isogenic *SF3B1* mutant clones

Flow cytometry plots showing the PE5max K700E electroporation efficiency and population targeted for sorting for A) HG-3 and B) MEC-1. Workflows for enriching the GFP bright population in C) HG-3 and D) MEC-1 cells sorted for PE5max K700E. Allelic discrimination plots showing the rhAMP SNP screening of single cell clones of E) HG-3 and F) MEC-1 cells sorted for GFP bright cells. For all allelic discrimination plots, the square boxes indicate allelic reference controls: K/K (blue) is homozygous WT using K562 *SF3B1* WT gDNA; K/E (green) is heterozygous mutant using K562 *SF3B1* K700E gDNA; E/E (red) is homozygous mutant using pUC19-*SF3B1*-K700E plasmid.
