# Supplementary material for: Engineering Oncogenic Hotspot Mutations on SF3B1 via CRISPR-Directed PRECIS Mutagenesis
Source: Cancer Res Commun. 2024 Sep 24;4(9):2498–513. doi: 10.1158/2767-9764.CRC-24-0145 (PMC11421219; doi:10.1158/2767-9764.CRC-24-0145)
Supplement: Supplementary Figure 5 — The K700E reporter is removed via Cre-mediated recombinase treatment [file crc-24-0145_supplementary_figure_5_suppsf5.pdf]

Supplementary Figure 5

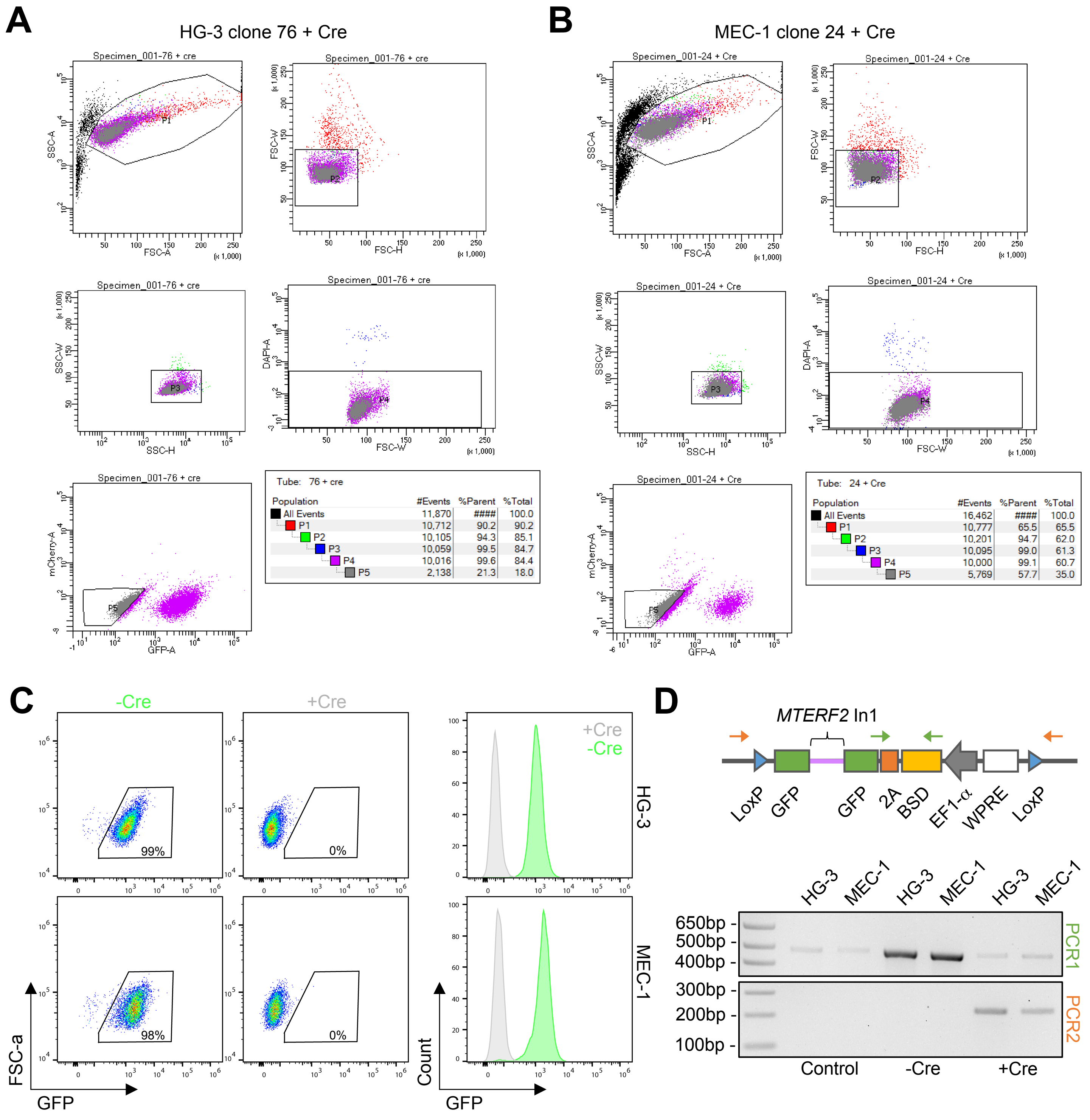

Supplementary Figure 5: The K700E reporter is removed via Cre-mediated recombinase treatment

Workflows for sorting out GFP negative cells following Cre overexpression for A) HG-3 and B) MEC-1 *SF3B1* K700E cells. C) Flow cytometry plots (left) and histograms (right) showing GFP expression with and without Cre overexpression in HG-3 and MEC-1 *SF3B1* K700E cells. D) (top) Primer designs and (bottom) PCR to genotype for deletion of the K700E reporter.
