# Supplementary material for: Engineering Oncogenic Hotspot Mutations on SF3B1 via CRISPR-Directed PRECIS Mutagenesis
Source: Cancer Res Commun. 2024 Sep 24;4(9):2498–513. doi: 10.1158/2767-9764.CRC-24-0145 (PMC11421219; doi:10.1158/2767-9764.CRC-24-0145)
Supplement: Supplementary Figure 6 — Splicing and growth phenotypes of HG-3 and MEC-1 SF3B1 mutant cell lines [file crc-24-0145_supplementary_figure_6_suppsf6.pdf]

Supplementary Figure 6

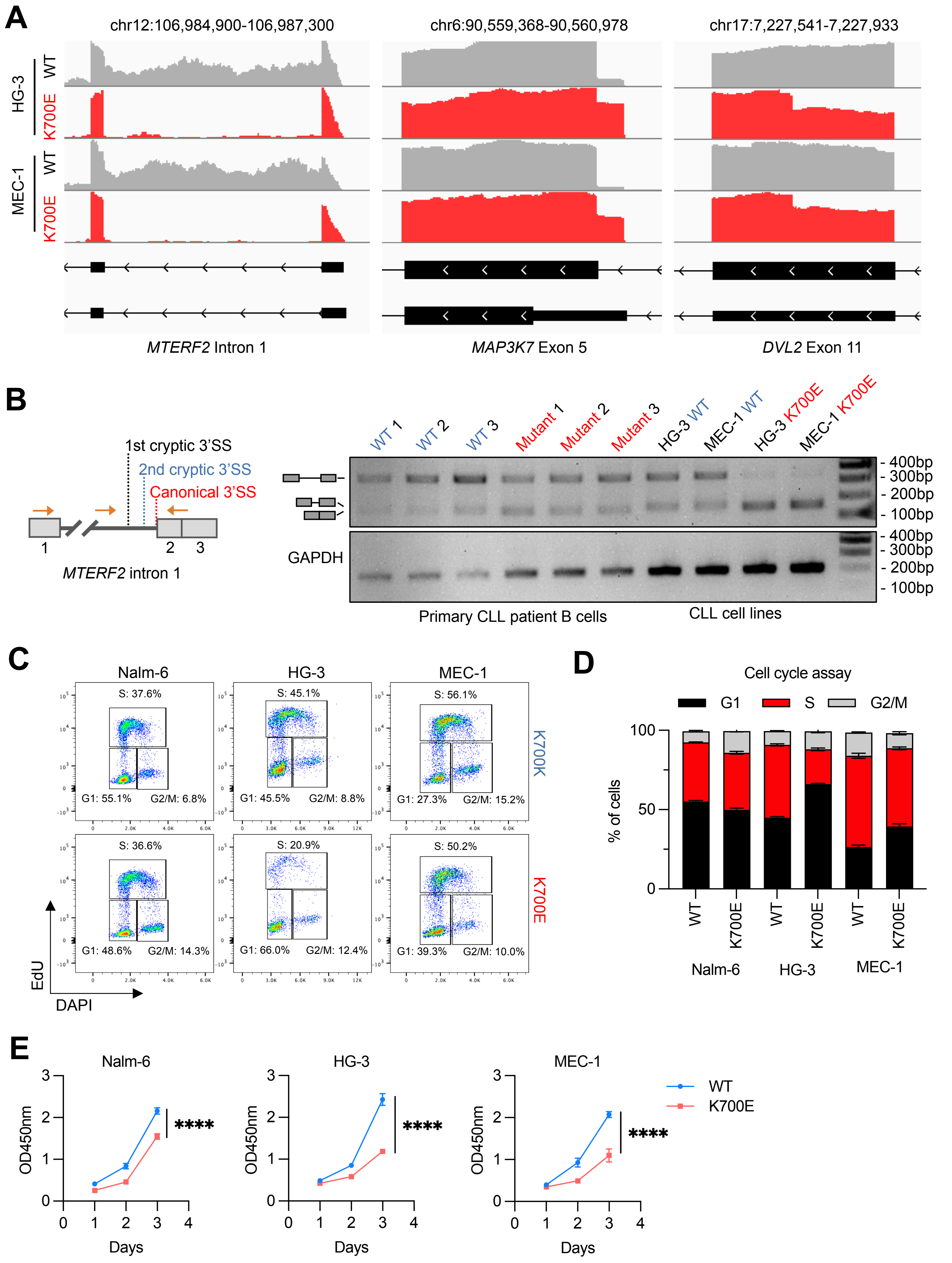

Supplementary Figure 6: Splicing and growth phenotypes of HG-3 and MEC-1 *SF3B1* mutant cell lines

A) RNA-seq analyses showing alternative splicing on *MTERF2*, *MAP3K7*, and *DVL2* between HG-3 and MEC-1 *SF3B1* WT and K700E cells. B) (top) Primer designs and (bottom) PCR for checking *MTERF2* intron 1 splicing in primary CLL patient samples versus CLL cell lines. C) Flow cytometry plots for the cell cycle analysis. D) Quantification of the results from D). Quantification was done on biological triplicates. E) CCK8 growth assays for *SF3B1* WT vs mutant cell lines in triplicates. Statistical test was done using two-way ANOVA (****$P \leq 0.0001$).
