# Supplementary material for: Engineering Oncogenic Hotspot Mutations on SF3B1 via CRISPR-Directed PRECIS Mutagenesis
Source: Cancer Res Commun. 2024 Sep 24;4(9):2498–513. doi: 10.1158/2767-9764.CRC-24-0145 (PMC11421219; doi:10.1158/2767-9764.CRC-24-0145)
Supplement: Supplementary Figure 7 — Aberrant 3’SS splicing profiles of SF3B1-mutated cell lines and primary CLL [file crc-24-0145_supplementary_figure_7_suppsf7.pdf]

# Supplementary Figure 7

A

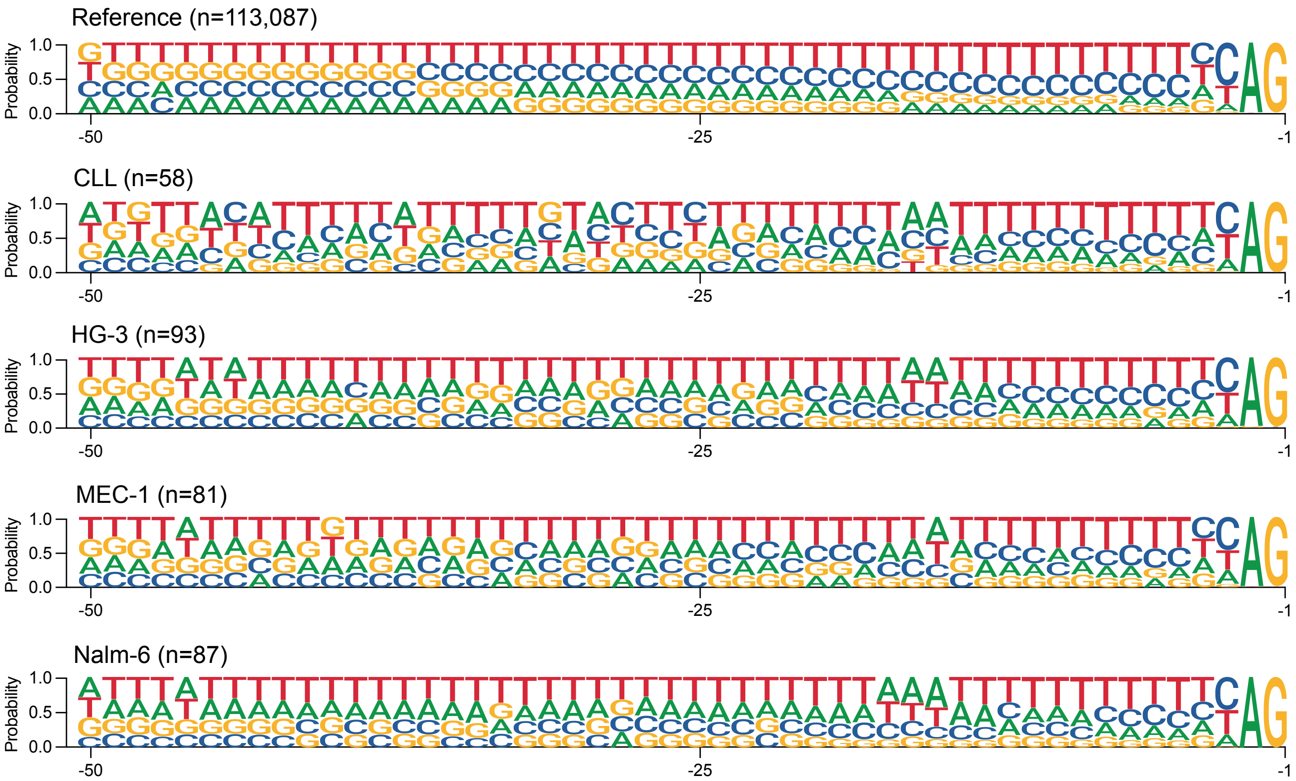


B

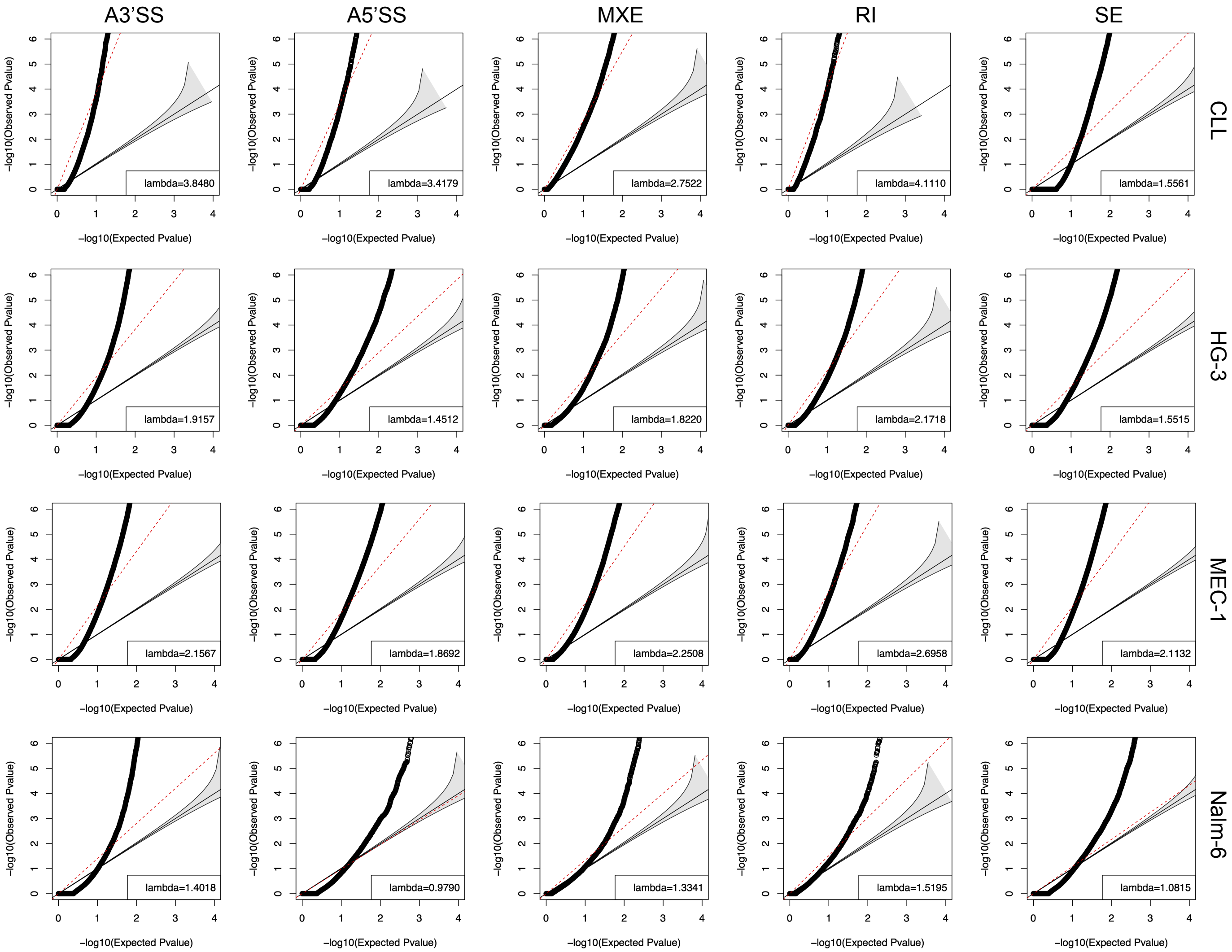

**Supplementary Figure 7: Aberrant 3'SS splicing profiles of *SF3B1*-mutated cell lines and primary CLL**

A) Logo plots showing the cryptic 3'SS sequence and location in *SF3B1*-mutated primary CLL samples and cell lines versus the RefSeq reference. The number of cryptic 3'SS events used to calculate each logo is indicated. B) Q-Q plots of observed P values versus expected P values for five types of alternative splicing events in *SF3B1*-mutated primary CLL samples and cell lines. Least-squares linear fit with slope $\gamma$ for the lower $95^{th}$ percentile is indicated by red lines. 95% confidence intervals are represented by the gray-shaded area.
