# Supplementary material for: Engineering Oncogenic Hotspot Mutations on SF3B1 via CRISPR-Directed PRECIS Mutagenesis
Source: Cancer Res Commun. 2024 Sep 24;4(9):2498–513. doi: 10.1158/2767-9764.CRC-24-0145 (PMC11421219; doi:10.1158/2767-9764.CRC-24-0145)
Supplement: Supplementary Figure 8 — RNAseqCNV analysis uncovers widespread CNV events in SF3B1 mutant cell lines [file crc-24-0145_supplementary_figure_8_suppsf8.pdf]

Supplementary Figure 8

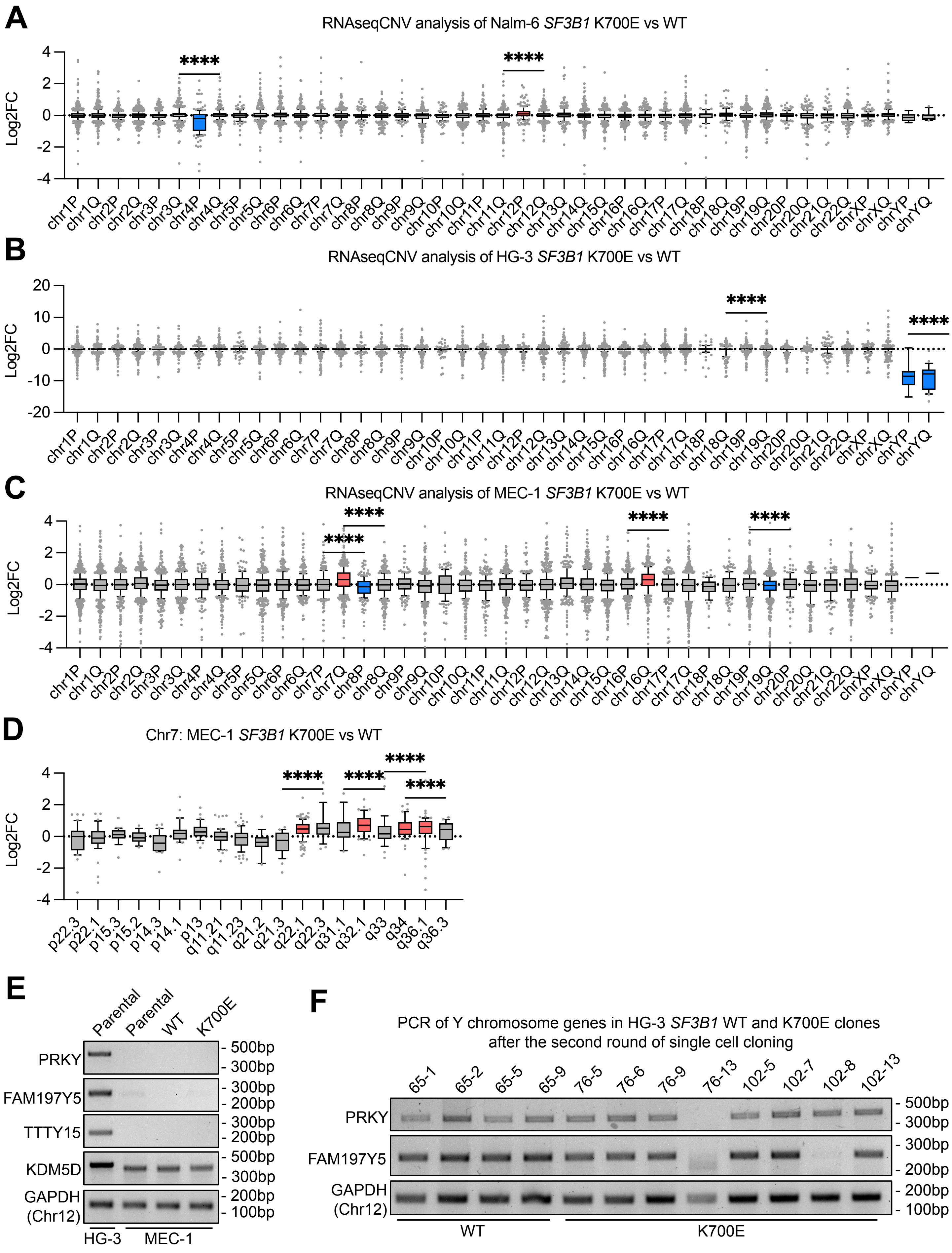

Supplementary Figure 8: RNAseqCNV analysis uncovers widespread CNV events in *SF3B1* mutant cell lines

RNAseqCNV analysis represented by bar plot based on gene expressions at each indicated chromosomal loci for A) Nalm-6, B) HG-3, and C) MEC-1. Regions that underwent amplification and deletion are shown in red and blue, respectively. Only chromosomal locations that have passed the one sample Wilcoxon signed rank test with a high statistical cutoff (****P≤0.0001) were annotated for deletion or amplification. D) Bar plot of gene expressions on chromosome 7. A chi-squared test was performed with $-\log_{10}(0.05)$ as the statistical cutoff. PCR of Y chromosome genes in the genomic DNA of E) parental and isogenic *SF3B1* WT and mutant cell lines and F) *SF3B1* WT and mutant cell lines after second round of single cell cloning at D90.
