# Supplementary material for: Engineering Oncogenic Hotspot Mutations on SF3B1 via CRISPR-Directed PRECIS Mutagenesis
Source: Cancer Res Commun. 2024 Sep 24;4(9):2498–513. doi: 10.1158/2767-9764.CRC-24-0145 (PMC11421219; doi:10.1158/2767-9764.CRC-24-0145)
Supplement: Supplementary Figure 9 — Off-targeting analyses for PRECIS engineering of SF3B1 K700E mutation [file crc-24-0145_supplementary_figure_9_suppsf9.pdf]

Supplementary Figure 9

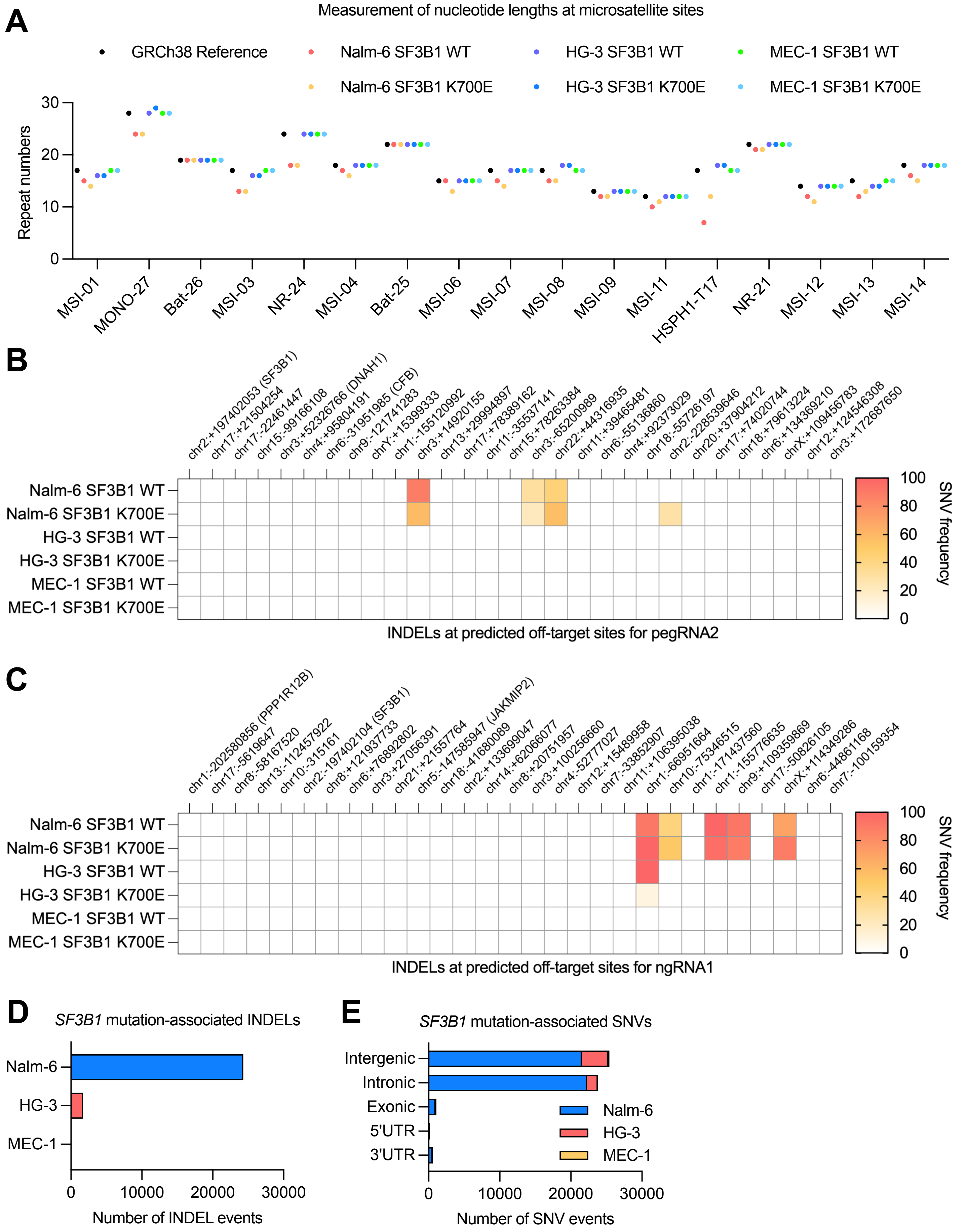

Supplementary Figure 9: Off-targeting analyses for PRECIS engineering of *SF3B1* K700E mutation

A) WGS measurements of repeat lengths at 17 microsatellite sites in isogenic cell lines versus the Hg38 reference genome. WGS measurement of INDEL frequencies at 29 predicted off-target sites for B) pegRNA2 and C) ngRNA1. D) Bar plot for number of INDEL events found in *SF3B1* mutant versus WT cell lines. E) Bar plot for the number of SNV events at different regions in the genome in *SF3B1* mutant versus WT cell lines.
