# Supplementary material for: Engineering Oncogenic Hotspot Mutations on SF3B1 via CRISPR-Directed PRECIS Mutagenesis
Source: Cancer Res Commun. 2024 Sep 24;4(9):2498–513. doi: 10.1158/2767-9764.CRC-24-0145 (PMC11421219; doi:10.1158/2767-9764.CRC-24-0145)
Supplement: Supplementary Table 1 — List of current SF3B1 mutant cell lines [file crc-24-0145_supplementary_table_1_suppst1.docx]

Supplementary Table 1: List of current *SF3B1* mutant cell lines

| Cell Line | Method | Mutation | Disease | Source |
| --- | --- | --- | --- | --- |
| HEK293T | Cas9 HDR | K666T | Human Kidney Fibroblast | Alsafadi et al, *Nature Communications* 2016 |
| HEK293T | Cas9 HDR | K700E | Human Kidney Fibroblast | Cusan et al, *Journal of Clinical Investigation* 2023 |
| MCF-10A | Cas9 HDR | K700E | Breast Cancer | Liu et al, *Journal of Clinical Investigation* 2020 |
| MCF-10A | AAV HDR | K700E, R702R | Breast Cancer | Dalton et al, *Journal of Clinical Investigation* 2019 |
| hTERT-IMEC | AAV HDR | K700E, R702R | Mammary Epithelial Cells | Dalton et al, *Journal of Clinical Investigation* 2019 |
| CCE mESC | Cas9 HDR | K700E | Mouse Embryonic Stem Cells | Gupta et al, *Nucleic Acids Research* 2018 |
| B16F10 | Cas9 HDR | R1074H | Mouse Melanoma Cells | Chang et al, *Journal of Biological Chemistry* 2021 |
| K-562 | AAV HDR | K700E | Chronic Myelogenous Leukemia | Dharman et al, *Cell Reports* 2015 |
| K-562 | Cas9 HDR | K700E | Chronic Myelogenous Leukemia | Liberante et al, *Scientific Reports* 2019 |
| K-562 | AAV+Cas9 HDR | K700E | Chronic Myelogenous Leukemia | Boddu et al, *Communication Biology* 2021 |
| K-562 | Cas9 HDR | K700E | Chronic Myelogenous Leukemia | Mian et al, *Science Translational Medicine* 2023 |
| Nalm-6 | AAV HDR | H662Q, K666N, K700E | Acute Lymphoblastic Leukemia | Dharman et al, *Cell Reports* 2015 |
| MEC-1 | Cas9 HDR | K700E | Chronic Lymphocytic Leukemia | Lopez-Oreja et al, *Life Science Alliance* 2023 |
